# An Optimized Workflow to Generate and Characterize iPSC-Derived Motor Neuron (MN) Spheroids

**DOI:** 10.3390/cells12040545

**Published:** 2023-02-08

**Authors:** María José Castellanos-Montiel, Mathilde Chaineau, Anna Kristyna Franco-Flores, Ghazal Haghi, Dulce Carrillo-Valenzuela, Wolfgang E. Reintsch, Carol X.-Q. Chen, Thomas M. Durcan

**Affiliations:** The Neuro’s Early Drug Discovery Unit (EDDU), McGill University, 3801 University Street, Montreal, QC H3A 2B4, Canada

**Keywords:** iPSC, motor neurons, spheroids, 3D model, motor neuron disease, MEA, CUBIC

## Abstract

A multitude of in vitro models based on induced pluripotent stem cell (iPSC)-derived motor neurons (MNs) have been developed to investigate the underlying causes of selective MN degeneration in motor neuron diseases (MNDs). For instance, spheroids are simple 3D models that have the potential to be generated in large numbers that can be used across different assays. In this study, we generated MN spheroids and developed a workflow to analyze them. To start, the morphological profiling of the spheroids was achieved by developing a pipeline to obtain measurements of their size and shape. Next, we confirmed the expression of different MN markers at the transcript and protein levels by qPCR and immunocytochemistry of tissue-cleared samples, respectively. Finally, we assessed the capacity of the MN spheroids to display functional activity in the form of action potentials and bursts using a microelectrode array approach. Although most of the cells displayed an MN identity, we also characterized the presence of other cell types, namely interneurons and oligodendrocytes, which share the same neural progenitor pool with MNs. In summary, we successfully developed an MN 3D model, and we optimized a workflow that can be applied to perform its morphological, gene expression, protein, and functional profiling over time.

## 1. Introduction

Motor neurons (MNs) are a subset of efferent neurons within the nervous system responsible for innervating the muscles of the body, promoting their contraction through highly specialized and structurally organized synapses termed neuromuscular junctions (NMJs) [1]. When the connection between MNs and skeletal muscle deteriorates or becomes interrupted as a result of MN degeneration, it leads to the development of a number of disorders known as motor neuron diseases (MNDs), which include amyotrophic lateral sclerosis (ALS), the most common disease in this category [2,3]. Recently, an epidemiologic study estimated ~260,000 individuals living with MNDs worldwide in 2019 [3]. Additionally, the analyzed data revealed that the global burden of MNDs is continuously increasing [3], highlighting the urge to develop new therapies for these diseases, which currently have no cure.

Animal models have been widely used to elucidate the disease mechanisms behind MNDs. However, rising doubts about the translatability of animal models to human patients due to failures in clinical trials, ethical concerns, and other disadvantages [4,5] have promoted the parallel development of in vitro models to increase our knowledge of MNDs and accelerate drug discovery [4]. With the advent of induced pluripotent stem cell (iPSC) technology, new approaches have emerged to generate and culture different cell types of the human body in vitro, including MNs [6]. Human iPSCs are generated by expressing the Yamanaka’s factors in adult somatic cells such as skin fibroblasts (FBs) or peripheral blood mononuclear cells (PBMCs) [7,8]. Thus, iPSCs can be derived from healthy individuals or patients with MNDs. In a disease context, patient-specific iPSCs retain the genetic background of the patient and, when differentiated into MNs, they can display characteristics of the diseases in vitro [9,10]. Remarkably, in cases where a monogenic mutation is identified as the primary cause of the disease, CRISPR-Cas9 genome editing can be used to correct the mutation, generating isogenic controls that facilitate precise genotype and phenotype correlations [11,12,13,14]. In addition to the latter advantage, iPSC-derived neurons allow researchers to study sporadic MND cases [9], which has never been achieved with other model systems. Taken together, these advantages open up new insights and therapeutic avenues to explore.

Most protocols available to generate iPSC-derived MNs are typically optimized to obtain two dimensional (2D) monolayer cultures [15,16,17,18,19,20], which have allowed the study of pathogenic molecular mechanisms associated with MNDs such as cytoskeletal abnormalities, axonal transport deficits, and changes in excitability [21,22]. However, several lines of evidence suggest that the absence of three-dimensional (3D) cell-to-cell and cell-to-matrix interactions may be detrimental to the maturation [23,24,25], differentiation [26], and morphology [27,28] of cells grown in vitro. In addition, technical challenges arise when culturing iPSC-derived MNs as 2D monolayers. For instance, the cell bodies of the MNs often form clusters, making them unsuitable for some assays [29,30,31] such as microelectrode array (MEA) analyses, which become challenging due to MNs randomly clustering away from the recording electrodes. Moreover, the clustering cells are prone to detachment from the surface [31], preventing their long-term culture and making the damaged floating monolayer of cells difficult to analyze by immunofluorescent staining or MEA. Maintaining cultures over several weeks is a critical aspect in MND modelling considering that disease phenotypes might only appear after a long period of culture. While some groups are optimizing 2D protocols to reduce the aggregation of iPSC-derived MNs [29,32], other groups are developing techniques to generate 3D cultures that include spinal organoids [33,34,35,36] and MN spheroids [25,37,38,39] to address some of the technical issues that arise with 2D monolayers and to enhance MN maturation by exposing the cells to a 3D environment. In fact, such 3D in vitro cultures have been proposed as an alternative to bridge the gap between current pre-clinical animal models and human studies given their greater physiological relevance compared to conventional monolayer cultures [5]. Within 3D in vitro models, spheroids have the advantage of being scaffold-free systems, opposed to organoid cultures, whose formation relies on the use of natural or synthetic matrices [40]. Typically, Matrigel is the preferred matrix for organoid generation, however, it is known to have high lot-to-lot variability that can impact culture results [40,41]. In addition, spheroids are smaller in size compared to organoids, making them less prone to some of the technical issues associated with 3D cultures such as the heterogenous distribution of components (i.e., oxygen and nutrients) [40]. Finally, there is no need for expensive and highly specialized equipment to maintain spheroids in culture, making them more cost effective than organoid approaches [40].

MN spheroids can be generated by making modifications to previously established 2D protocols [25,37,38,39,42]. Plating neural stem cells (NSCs) [25,37,43], motor neuron progenitor cells (MNPCs) [43], or MNs [38,39] onto a low-attachment surface takes advantage of their ability to spontaneously assemble into clusters and form spheroids in which they can continue their differentiation towards MNs. Even though these MN spheroids show expression of well-known MN markers (i.e., pan-MN marker motor neuron and pancreas homeobox 1/ISL LIM homeobox 1 (HB9/ISL1) and choline acetyltransferase (CHAT)), a characterization at the gene expression and protein levels using different markers to identify the presence of other cell types such as interneurons (INs) and glial cells within MN spheroids is lacking. Additionally, the development and optimization of a defined workflow to analyze these 3D structures in more detail is a challenging task that needs to be addressed. With this in mind, we adapted a previously established 2D protocol [44] to generate 3D MN spheroids from iPSC-derived MNPCs that can be grown and maintained for up to 28 days, and longer if needed. By applying tissue-clearing, 3D optical imaging, and quantitative real-time PCR (qPCR), we characterized the identity of the cells within the MN spheroids and found that the majority of the cells expressed MN markers. Moreover, by performing microelectrode array (MEA) activity recordings, we confirmed that these MN spheroids contain electrically-active neurons.

The differentiation process from iPSCs towards a desired cell type can be time-consuming. Thus, a technical advantage associated with the selected protocol for MNPC generation is that it allows for their storage and expansion [12,16]. Importantly, we showed that these MNPCs retain their capacity to proliferate and form MN spheroids across passage number, saving time for the end user and increasing the cost-effectiveness of the process. This work describes the optimization of several techniques that can help bridge the gap of information observed in similar studies. For instance, our protocol showed that optical opacity, often portrayed as a disadvantage of 3D models [40], can be easily overcome in this type of model by performing a tissue-clearing protocol that preserves the cytoarchitecture of the samples and enables the visualization of proteins by immunocytochemistry using conventional fluorescence microscopy. Moreover, we outline our workflow to obtain activity readouts from MN spheroids, which are not suitable for classic patch-clamp analysis [40]. All together, we develop a new, reliable, and efficient pipeline to characterize MN spheroids that has the potential to be applied to other types of neural spheroids. Easy to maintain and generated at high numbers, spheroids represent a simple model that has the potential to be applied to different assays and to be used as a cellular jigsaw, in which other cell types can be added to form more advanced co-culture spheroids.

## 2. Materials and Methods

### 2.1. Generation and Maintenance of Human iPSCs

Two control iPSC lines were used to optimize the protocols constituting the workflow for this study: AIW002-02 and 3450. The complete profiles of the iPSCs, culture conditions, and quality control analysis have been published [12,45,46]. The 3450 and AIW002-02 iPSCs were thawed and passaged in Matrigel-coated dishes. To choose the ideal culture medium, iPSCs were cultured in E8 and mTeSR1 media. The medium promoting the fastest growth rate of the cells without having an impact on their overall attachment, spontaneous differentiation, and morphology was selected to maintain each iPSC line. The 3450 iPSC line exhibited a faster growth rate when cultured in E8 medium, while the AIW002-02 iPSC line exhibited a faster growth rate when cultured in AIW002-02 medium [45]. Both iPSC lines were between passage 8 and 12 after performing the reprogramming and quality control steps. For every independent differentiation, iPSCs were kept under passage 15. Genomic stability testing at different passages through karyotyping and qPCR has shown that exceeding 15 passages can lead to the appearance of genomic alterations [45]. Prior to the experiments, the iPSCs were found to be free from mycoplasma, hepatitis B/C, and HIV 1/2 virus.

### 2.2. Generation of iPSC-Derived MN Spheroids

Starting from iPSCs, we adapted a previously described protocol [12,16] to derive MNPCs (Supplementary Figure S1) that can be used to generate MN spheroids. The media and biochemicals used to generate MN spheroids are listed in Table 1. Consumables and equipment are listed in Supplementary Table S1. The media compositions for the different differentiation steps are listed in Table 2.

#### 2.2.1. Day 0 (D0): Seeding of iPSCs

Start from a 100 mm dish of human iPSCs with a confluence between 70 and 80%, containing less than 5% of spontaneously differentiated cells. Once cells are ready, aspirate the medium from the iPSC culture and wash with 5 mL of DMEM/F-12 containing 1X Antibiotic-Antimycotic (Anti-Anti). Add 5 mL of Gentle Cell Dissociation Reagent (GCDR) and incubate at room temperature (RT) for 4–5 min. Avoid incubating the iPSCs in GCDR for too long, as cells dissociated into single cells are not ideal for the induction process. Aspirate the GCDR and rinse the cells with 5 mL of DMEM/F-12 containing 1X Anti-Anti. Aspirate the medium and then add 5 mL of DMEM/F-12 containing 1X Anti-Anti. Gently detach the colonies with a cell scraper and transfer the cells to a 15 mL conical tube. Wash the dish with an additional 5 mL of DMEM/F-12 containing 1X Anti-Anti to collect the remaining detached cells. Determine the cell number using a cell counter. Transfer 2–3 million cells to a fresh 15 mL conical tube and pellet the cells by centrifugation at 1200 rpm for 3 min. Aspirate the supernatant. Gently resuspend the cells in 1 mL of neural induction medium containing ROCK inhibitor (10 μM). Transfer the cells to a Matrigel-coated T-25 flask and add 4 mL of neural induction medium containing ROCK inhibitor (10 μM). Distribute the cells uniformly and place the flask in a 37 °C/5%CO_2_ incubator.

Note: The quality of the iPSCs [45] is crucial to the successful generation of MNPCs. Thus, mycoplasma tests should be performed routinely, ideally every other week, to confirm the absence of contamination before using the iPSCs. To verify the absence of mycoplasma from cultures, we used a mycoplasma detection assay (MycoAlert^TM^ mycoplasma detection kit, Lonza) to measure the luminescence of 1.5 mL medium samples, according to manufacturer’s instructions.

#### 2.2.2. Day 1 (D1): Neural Induction

Within 24 h of plating the iPSCs, change the medium to 5 mL of neural induction medium without ROCK inhibitor. Continue to change the medium every other day until D6 to induce the cells towards a neural progenitor cell (NPC) identity.

Check the cell morphology and density of the cells 24 h after plating. The cells must be 30% confluent. If the cells are too confluent, causing the medium to turn yellow, change the medium every day until D6.

#### 2.2.3. Day 6 (D6): MNPC Patterning

Six days after plating the iPSCs in neural induction medium, the cells should be 100% confluent and differentiated into NPCs. For the next step, cells will be plated on a T-25 and a T-75 PLO/laminin-coated flask. For PLO/laminin coating, PLO must be kept for at least 2 h, or overnight, then washed three times with 1X PBS and switched to laminin for a minimum of 2 h, or overnight. Aspirate the neural induction medium and rinse the cells with 5 mL of DMEM/F-12 containing 1X Anti-Anti. Add 2 mL of GCDR and incubate at 37 °C for 5–7 min. After the incubation, cells will begin lifting from the dish. Gently tap the plate to allow for the complete detachment of the cells into the GCDR. Add 5 mL of DMEM/F-12 containing 1X Anti-Anti and transfer the cells to a 15 mL conical tube. Pellet the cells by centrifugation at 1200 rpm for 3 min. Aspirate the supernatant. Resuspend the cells in 4 mL of MNPC patterning medium containing ROCK inhibitor (10 μM) and gently pipette the cells up and down. Plate 3 mL of the cell suspension into the PLO/laminin-coated T-75 flask. Complete with 12 mL of MNPC patterning medium containing ROCK inhibitor (10 μM) to reach a final volume of 15 mL and distribute the cells uniformly. Plate the remaining mL of the cell suspension into the PLO/laminin-coated T-25 flask. Complete with 4 mL of MNPC patterning medium containing ROCK inhibitor (10 μM) to reach a final volume of 5 mL and distribute the cells uniformly. Place the flasks in a 37 °C/5%CO_2_ incubator and, 24 h after plating the NPCs, change the medium to MNPC patterning medium without ROCK inhibitor. Continue to change the MNPC patterning medium every other day until D12.

Check the cell morphology and density of the cells 24 h after plating. The cells must be 50% confluent. If the cells are too confluent, causing the medium to turn yellow, change the medium every day until D12.

#### 2.2.4. Day 12 (D12): MNPC Expansion

Six days after plating the NPCs in MNPC patterning medium, cells should be 70% to 100% confluent and patterned to MNPCs (Supplementary Figure S1). For the next step, cells will be plated into four T-75 PLO/laminin-coated flasks. Split the cells following Section 2.2.3. Resuspend the cells in 4 mL of MNPC expansion medium containing ROCK inhibitor (10 μM) and gently pipette the cells up and down. Plate 1 mL of cell suspension into each of the four PLO/laminin-coated T-75 flasks. Complete with 14 mL of MNPC expansion medium containing ROCK inhibitor (10 μM) to reach a final volume of 15 mL and distribute the cells uniformly. Place the flasks in a 37 °C/5% CO_2_ incubator. At 24 h after plating the MNPCs, change the medium to MNPC expansion medium without ROCK inhibitor. Continue to change the medium every other day until D18.

If the cells are too confluent, causing the medium to turn yellow, change the medium every day until D18.

#### 2.2.5. Day 18 (D18): MNPC Storage, Split, or MN Spheroid Generation

MNPC storage: Six days after plating the MNPCs in MNPC expansion medium, cells should be 70% to 100% confluent. Proceed directly to freeze or split the cells. MNPCs can be frozen up to passage 3, depending on the cell morphology and density of the cells. MNPCs reduce their proliferation after 4 or more passages.

The 4 T-75 flasks in MNPC expansion medium can be frozen into 20 to 40 cryovials, with each vial containing ~3–5 million cells in 1 mL of FBS containing 10% DMSO.

MNPC thawing: Thaw the frozen cryovial of MNPCs in a 37 °C water bath by gently shaking the cryovial continuously until only a small, frozen cell pellet remains. Sterilize the outside of the cryovial with 70% ethanol. Transfer the cells to a 15 mL conical tube with 4 mL of DMEM/F-12 containing 1X Anti-Anti and pipette gently. Pellet the cells by centrifugation at 1200 rpm for 3 min. Aspirate the supernatant. Resuspend the cells in 5 mL of MNPC expansion medium containing ROCK inhibitor (10 μM) and transfer them into a T-25 PLO/laminin-coated flask. Place the flask in a 37 °C/5% CO_2_ incubator. At 24 h after plating the MNPCs, change the medium to MNPC expansion medium without ROCK inhibitor. Continue to change the MNPC expansion medium every other day for 6–7 days.

MNPC split: Before starting MN spheroid generation from a recently thawed MNPC cryovial, it is recommended to passage the cells at least once to allow their complete recovery. After each passage, plate two T-25 flasks. One flask will be used to keep the MNPC stock in culture (up to five passages) through GCDR dissociation, and the other flask will be used to initiate MN spheroid generation through Accutase dissociation. Split the cells following Section 2.2.3. Resuspend the cells in MNPC expansion medium containing ROCK inhibitor (10 μM) and plate ~3–4 million cells into a PLO/laminin-coated T-25 flask. Complete with MNPC expansion medium containing ROCK inhibitor (10 μM) to reach a final volume of 5 mL and distribute the cells uniformly and place the flask in a 37 °C/5% CO_2_ incubator. Within 24 h of plating the MNPCs, change the medium to MNPC expansion medium without ROCK inhibitor. Continue to change the MNPC expansion medium every other day for 6–7 days.

MN spheroid generation: MNPCs are ready for differentiation when cells have been cultured in MNPC expansion medium for 6–7 days. Split the cells following Section 2.2.3, incubating with 2 mL of Accutase at 37 °C for 4–6 min instead of GCDR to achieve a single cell suspension. Monitor the cells so the dissociation can be stopped as soon as all cells have detached. Resuspend the cells in 1 mL of MN induction and maturation medium without ROCK inhibitor and pipette the cells up and down. Quantify cell number using a cell counter. Plate 5000 MNPCs in 100 μL into each well of a 96 U-bottom ultra-low attachment plate in MN induction and maturation medium. Centrifuge the plate at 1200 rpm for 5 min using a centrifuge with adapters for cell culture plates. Place the culture plate in a 37 °C/5% CO_2_ incubator. This centrifugation step will speed up the aggregation of the cells, thus speeding up the formation of the spheroids. However, the spheroids will also form (albeit at a slower rate) even if this step is not performed. To maintain the MN spheroids in culture over time, replenish each well of the 96 U-bottom ultra-low attachment plate with 50 μL of MN induction and maturation medium every 14 days.

Note: When plating the cells into the 96 U-bottom ultra-low attachment plate, avoid seeding cells in the outer wells of the plate. Evaporation of the medium is higher in these wells and it has a negative impact on spheroid formation. Instead, fill the outer wells with 200 μL of 1X PBS. Although rare, we have noticed random inappropriate formation of MN spheroids regardless of the iPSC line (Supplementary Figure S2). Instead of fusing into a single spheroid, MNPCs formed two or more spheroids. In this case, those spheroids were not considered for any further analysis.

### 2.3. Cell Profiler Macro for Size Profiling of MN Spheroids

The equipment used to profile the size of the MN spheroids is listed in Supplementary Table S1. Bright-field images of MN spheroids were acquired with a light microscope after culture for 14 and 28 days in MN induction and maturation medium. Importantly, the images acquired at the two different time points were always saved with their location within the plate (Ex. B2, B3, B4, B5…) in order to follow each MN spheroid growth over time. We performed measurements on 28–30 MN spheroids from each of five different batches for each cell line (AIW002-02 = 150 spheroids; 3450 = 145 spheroids). The images were analyzed by a modified version of a pipeline developed in our group using CellProfiler Analyst, www.cellprofiler.org (accessed on 1 August 2021) [46,49], available online (https://doi.org/10.17605/OSF.IO/V84WS) (accessed on 27 September 2021). Briefly, images of the spheroids obtained with a bright-field microscope are inverted, and speckles with a diameter smaller than 10 pixels are filtered out to eliminate cell debris and cells that were not incorporated into the spheroids. From this point, the primary object (named “Sphere”) is identified using Otsu thresholding and its size and shape are measured (Supplementary Figure S3A). The primary objects identified as the final spheroids are overlaid with the original input image for visual control of the spheroid identification (Supplementary Figure S3B). As a result, the pipeline gives an CSV file with different measurements in pixels for each MN spheroid, including the min. Feret diameter (used as diameter) and the area. The measurements in pixels have to be manually transformed into μm (diameter) or μm^2^ (area) according to the microscope scale if needed. The radius of each spheroid was calculated by dividing the min. Feret diameter by half and substituting it into the formula (4/3∏r^3^) to determine the volume of a sphere. In addition, to assess the circularity of the spheroids, the radius of each spheroid was substituted into the formula (∏r^2^) to determine the area of a circle, and a ratio between the calculated area and the area given by the software was performed. The efficiency of the CellProfiler pipeline to identify the primary objects (spheroids) highly depends on the pixel intensities through the entire bright-field image, meaning that sometimes cell debris or individual cells with a pixel intensity similar to the sphere lead to misinterpretation of the primary object (Supplementary Figure S3C). To establish a threshold that helped us define images in which the CellProfiler pipeline performed poorly, we used GraphPad Prism (created by Dotmatics, version 9.1.1, Boston, MA, USA) to identify outliers using the ROUT method with a Q = 1%. The images marked as potential outliers were checked to corroborate that they were not the actual size of the spheroid. A paired t-student was performed to compare the diameter, area, volume, and circularity measurements of 14- and 28-day MN spheroids (AIW002-02 = 137 spheroids; 3450 = 142 spheroids).

### 2.4. qPCR Analysis of MN Spheroids

MN spheroids from one 96 U-bottom ultra-low attachment plate (60 spheroids) were pooled at 14 and 28 days for RNA extraction. The reagents to perform the qPCR from MN spheroid samples are listed in Table 3. The consumables and equipment are listed in Supplementary Table S1. The probes used for the qPCR analysis are listed in Table 4.

Total RNA was isolated using the miRNeasy micro kit according to manufacturer’s instructions. After RNA isolation, reverse transcriptions were performed to obtain cDNA. For each sample, 40 ng of total RNA was diluted in distilled water to reach a final volume of 10 µL. Next, 16 µL of M1 mix (distilled water, 0.5 mM dNTPs, and 12.5 ng/μL of random primers) was added to each reaction tube. A denaturation step was performed in the thermocycler at 65 °C for 5 min on the total RNA mixed with the M1 mix, constituting a volume of 26 µL. Finally, 14 µL of M2 mix (0.01 M DTT, 1X first strand buffer and 400 U M-MLV RT) was added to each reaction tube to make a total volume of 40 μL. The reverse transcriptions were performed with an incubation at 37 °C for 50 min followed by an incubation at 70 °C for 10 min in the thermocycler. qPCR reactions were performed in 384-well plates using the QuantStudio5 PCR machine. For each well, the PCR mix included 9 μL of 2X no UNG Taqman ^TM^ Fast Advanced Master, 0.5 μL of primers/probe mix, 1 μL of cDNA, and H_2_O up to 10 μL. Serial dilutions of a mix of cDNA, consisting of cDNA from all the samples, ranging between 50 ng and 0.003052 ng, were used to generate a calibration curve for absolute quantification [12,50,51,52]. Expression levels were given as a ratio between the relative quantities of the gene of interest and the endogenous control. The mean between Actβ and GAPDH was used as the endogenous control for normalization.

### 2.5. Fixation, Tissue Clearing, and Immunofluorescent Staining of MN Spheroids

MN spheroids from 96 U-bottom ultra-low attachment plates (60 spheroids/plate) were fixed, cleared, and immunostained at 14 and 28 days [46,53,54]. The reagents to perform the fixation and the immunofluorescent staining of the MN spheroids are listed in Table 5. The compositions to prepare the two solutions needed to perform the Clear Unobstructed Brain/Body Imaging Cocktail and Computational Analysis (CUBIC) protocol [53], CUBIC reagent 1 (R1) and CUBIC reagent 2 (R2), are listed in Table 6. The composition of the blocking solution used for the immunostaining process is listed in Table 7. The consumables and equipment are listed in Supplementary Table S1. The primary and secondary antibodies used for immunostaining are listed in Table 8.

A maximum of six MN spheroids per 0.6 mL collection tube were transferred from the 96 U-bottom ultra-low attachment plates, fixed in 4% FA for 15–20 min at RT and washed three times with 1X PBS. To perform all the PBS washes of Section 2.5., the spheroids were allowed to reach the bottom of the collection tube through gravity (~5 min) before removing the supernatant. For each wash, 400 μL of 1X PBS was used and the tube was left in the nutating mixer for 10 min. After fixation, the CUBIC protocol was performed by replacing 1X PBS with 200 μL of CUBIC R1. Incubation of the samples with CUBIC R1 was performed at 37 °C with gentle shaking (~80 rpm) for 48–72 h. The removal of CUBIC R1 was achieved by performing three 1X PBS washes. MN spheroids were blocked in 200 μL of blocking solution overnight at 37 °C with gentle shaking (~80 rpm). Blocking solution was removed by allowing the spheroids to reach the bottom of the collection tube by gravity (~5 min). Primary antibodies were diluted in blocking solution and added to the MN spheroids for 24–72 h at 37 °C with gentle shaking (~80 rpm). A final volume of 150 μL was used per collection tube. Primary antibodies were washed out by performing three 1X PBS washes. Secondary antibodies and Hoechst33342 were diluted in blocking solution and added to the MN spheroids for 24–72 h at 37 °C with gentle shaking (~80 rpm). A final volume of 150 μL was used per collection tube. Secondary antibodies and Hoechst33342 were washed out by performing three 1X PBS washes.

For imaging, we optimized a protocol to image MN spheroids at large scale using black 96-well plates. Each spheroid was transferred into the center of a well using wide-orifice low-binding tips. Excess 1X PBS was removed and 100 μL of CUBIC R2 was added per well as mounting medium. Images of the immunostained MN spheroids were acquired with the Opera Phenix High-Content Screening System using the PreScan function to find the spheres within the focal plane at 5X and then perform the imaging at 20X. System 5×/0.16 and 20×/1.0 objectives. Image size 512 × 512, voxel size 0.29 × 0.29 × 5 µm. The data were extracted to be organized and analyzed by an in-house script developed in MATLAB. Images were analyzed as raw Z-stacks without altering brightness and contrast. Alternatively, MN spheroids can be mounted over a glass slide to be imaged with a conventional confocal microscope. For this, a hydrophobic pen is used to draw a circle in the middle of a glass slide and an MN spheroid is transferred in the center using wide-orifice low-binding tips. Excess 1X PBS was removed and a drop of CUBIC R2 (~30 μL) was added to the spheroids, followed by the placing of a glass coverslip on top.

**Note:** CUBIC R1 and R2 solutions must be filtered using a cell strainer to remove any solutes that did not incorporate into the solution. This will reduce undesired detritus at the imaging step. After the addition of CUBIC R1, the spheroids become transparent, and they are almost imperceptible to the eye. After the first 1X PBS wash, the spheroids recover their white color, and it is possible to visualize them again. For the mounting process inside black 96-well plates, it is essential to remove almost all the 1X PBS surrounding the spheroids before adding the CUBIC R2. This will prevent the spheroids from floating at different heights inside the well, thus making their tracing difficult at the microscope.

### 2.6. Microelectrode Array (MEA) Recordings of MN Spheroids

Each batch of MNPCs was used to generate one 96 U-bottom ultra-low attachment plate (60 spheroids). The reagents to perform MN spheroid MEA recordings are listed in Table 9. The consumables and equipment are listed in Supplementary Table S1. The composition of the artificial cerebrospinal fluid (aCSF) solution needed for MEA recordings is detailed in Table 10.

Cytoview 24-well MEA plates with 16 electrodes per well were treated with PLO (10 μg/mL) for 24 h, washed 3 times with 1X PBS, and coated with laminin (5 μg/mL) for 24 h. MN spheroids were generated and cultured for 7 days into the 96 U-bottom ultra-low attachment well plates before being transferred to each well of the Cytoview 24-well MEA plate using wide-orifice low-binding tips. Six whole spheroids were deposited in the center of each well [55] in no more than 20 μL of medium to prevent the spheroids from spreading to the edges of the well. The plate was returned to the incubator for 20–30 min to allow the spheroids to attach to the electrodes before addition of 500 μL of fresh MN induction and maturation medium.

Before each recording, 50 mL of 1X aCSF solution was prepared from a 10X stock solution, adding D-glucose and NaHCO_3_ at the working concentrations. The tube with the 1X aCSF solution was left with the lid loose inside the incubator for 1 h to equilibrate the solution with the same gaseous conditions found inside the incubator. Before the 1X aCSF solution was added to the cells, it was filtered to avoid any possible contamination. After removing the MN induction and maturation medium and adding 500 μL of the 1X aCSF solution to each well, the MEA plate was returned to the incubator at 37 °C/5% CO_2_ for 30–45 min.

MEA recordings were performed on days 7, 14, 21, and 28 after plating the MN spheroids. Data were collected for 5 min using the Axis Navigator software (provided by Axion Biosystems, version 1.5.1.12, Atlanta, GA, USA). A band-pass filter of 3 kHz (low-pass) to 200 Hz (high-pass) was applied. For the analysis, a “spike” was defined as a short extracellular electrical event with a peak voltage six times or greater than the standard deviation of the estimated “noise” signal. A “burst” was defined as ≥5 spikes with no more than 100 ms separating each spike. Network bursts were not measured, as the presence of spheres within the plate was random with very few spheres centered within an electrode for the entire recording period of 28 days. The MEA plate was placed into the Axion Maestro Edge with temperature and CO_2_ concentration set to 37 °C and 5%, respectively. The plate was allowed to equilibrate for 5 min inside the instrument prior to recording. After the recording, the MEA plate was removed from the instrument and the aCSF was replaced with MN induction and maturation medium to keep the cells in culture for the following recording time points. At day 28, after the basal recording of 5 min in aCSF was performed, the plate was removed from the instrument and dosed with vehicle (H_2_O) or 1 mM tetrodotoxin (TTX). The plate was returned to the instrument and allowed to equilibrate for 5 min before performing a second recording of 5 min. This was the endpoint of the MEA recordings.

## 3. Results

**Figure 1:**
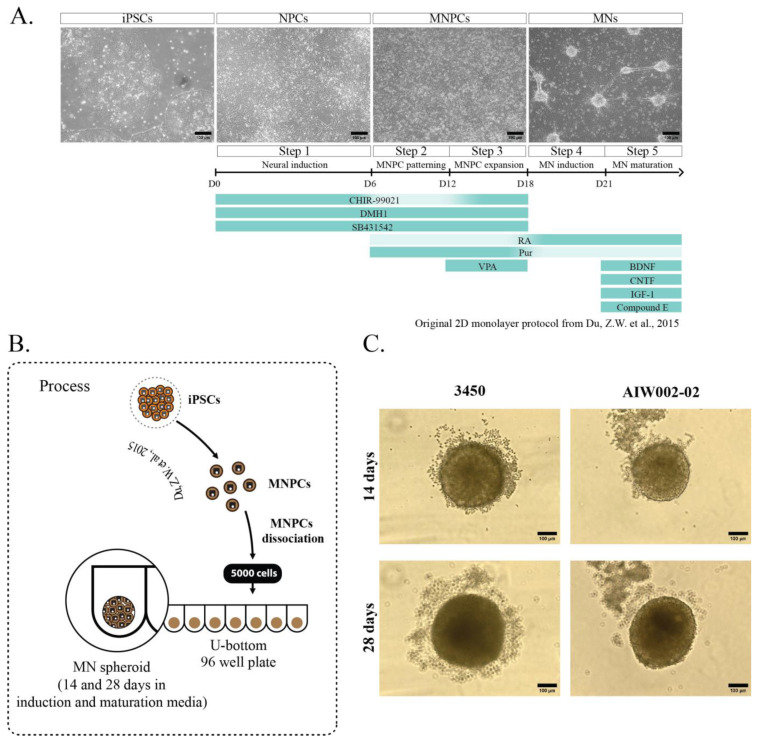
**Generation of iPSC-derived MN spheroids.** (**A**) Summary of the small-molecule protocol developed by [16] and optimized by our group to generate iPSC-derived MNs as a 2D monolayer. Briefly, iPSCs are differentiated towards neural progenitor cells (NPCs) that are patterned to motor neuron neural progenitor cells (MNPCs), which are finally differentiated into MNs. (**B**) At the MNPCs stage, two control cell lines (3450 and AIW002-02) were dissociated and plated into 96 U-bottom ultra-low attachment well plates in MN induction and maturation medium to generate MN spheroids that were kept in culture until their analysis. (**C**) Representative bright-field pictures of MN spheroids after 14 and 28 days of differentiation of both control cell lines.

**Figure 2:**
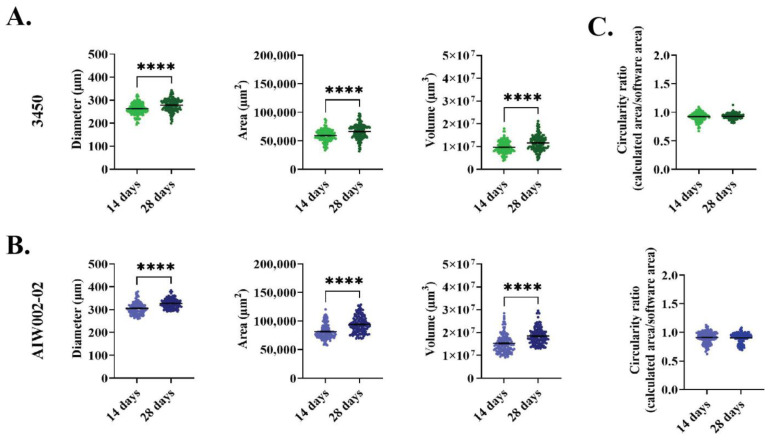
**Size profile by a cell profiler pipeline.** iPSC-derived MN spheroids from the (**A**) 3450 and (**B**) AIW002-02 control lines are consistently generated across different batches. They exhibit an increase in size over time as shown by diameter, area, and volume measurements. Scatter plots show the mean ± SEM; for each cell line, five MN spheroid batches coming from at least two iPSC-derived MNPCs batches induced through independent differentiation processes (AIW002-02 = 137 spheroids; 3450 = 142 spheroids). To ensure that MNPC passage number was not having any effect on MN spheroid formation, we used the MNPCs at passage number 2 to 4. Significance was determined by a paired *t*-student. **** *p* ≤ 0.0001. (**C**) Additionally, MN spheroids display a circular shape with bulges appearing occasionally but without disturbing the circular morphology, as shown by the circularity ratio (0 = not circular; 1 = perfect circle).

We generated MN spheroids from two different healthy control iPSC lines (AIW002-02 and 3450), adapting a previously described protocol used to generate MNs as a 2D monolayer (Figure 1A) [16]. For our experiments, 80% confluent iPSC cultures were differentiated into MNPCs. At the MNPC stage, we confirmed the expression of the neural precursor markers Sox1, Nestin, and Pax6 [56,57], in addition to Ki67, an endogenous marker of active cell cycle, thus confirming the cells’ proliferation capacity [58] (Supplementary Figure S1). MNPCs also co-expressed the markers Nkx6.1 and Olig2, confirming their identity as MNPCs [59] (Supplementary Figure S1). From these MNPCs, 5000 cells per well were used to generate iPSC-derived MN spheroids into 96-well U-bottom ultra-low attachment plates (Figure 1B). Diameter (3450, 14 days = 262.9 μm ± 1.976, 28 days = 278.3 μm ± 2.255; AIW002-02, 14 days = 305.4 μm ± 2.225, 28 days = 326.4 μm ± 1.752), area (3450, 14 days = 5.92 × 10^4^ μm^2^ ± 804.4, 28 days = 6.61 × 10^4^ μm^2^ ± 1065; AIW002-02, 14 days = 8.13 × 10^4^ μm^2^ ± 1090, 28 days = 9.40 × 10^4^ μm^2^ ± 1244), and volume (3450, 14 days = 9.73 × 10^6^ μm^3^ ± 214,272, 28 days = 1.15 × 10^7^ μm^3^ ± 274,041; AIW002-02, 14 days = 1.52 × 10^7^ μm^3^ ± 346,330, 28 days= 1.84 × 10^7^ μm^3^ ± 303,753) measurements were obtained by analyzing bright-field images of the MN spheroids (Figure 1C) with a CellProfiler Analyst pipeline. We observed that the spheroids from both control lines grew in size from 14 to 28 days, as confirmed by increases in the measurements of their overall diameter (3450 = *p* < 0.0001; AIW002-02 = *p* < 0.0001), area (3450 = *p* < 0.0001; AIW002-02 = *p* < 0.0001), and volume (3450 = *p* < 0.0001; AIW002-02 = *p* < 0.0001) (Figure 2A,B). Importantly, since our protocol allows us to expand the MNPCs for up to five passages [16], we generated MN spheroids from MNPCs between passages two and four, and we observed that the spheroids were successfully generated and kept their capacity to grow over time regardless of the passage number. In terms of morphology, the MN spheroids display a circular shape, however, an analysis of circularity (Figure 2C) showed that the spheroids are not perfectly round (or circularity ratio = 1). Taken together, we succeeded in the establishment of culture conditions to generate 3D iPSC-derived MN spheroids in a reproducible manner.

**Figure 3:**
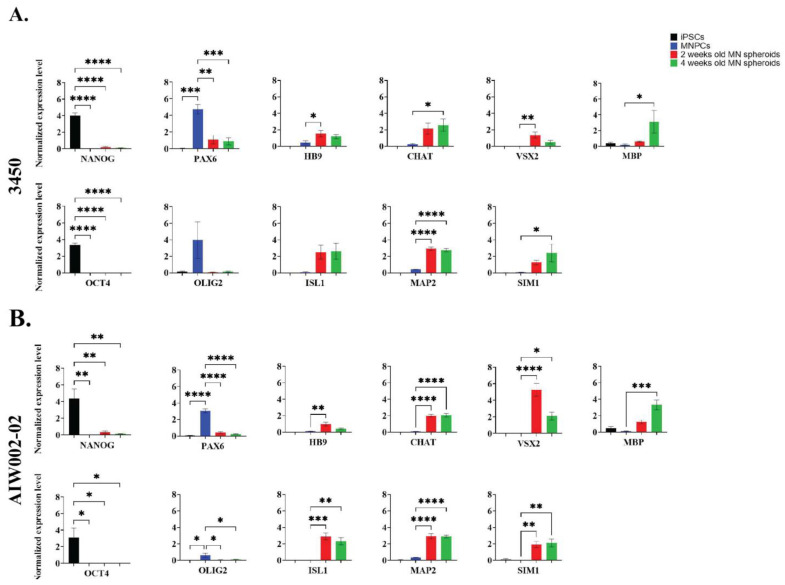
**Transcript expression profile of iPSC-derived MN spheroids.** Normalized expression levels of NANOG, OCT4, PAX6, OLIG2, HB9, ISL1, CHAT, MAP2, VSX2, SIM1, MBP, and GFAP in iPSCs, MNPCs, and iPSC-derived MN spheroids differentiated for 14 and 28 days from (**A**) 3450 and (**B**) AIW002-02 lines. Data normalized to Actβ-GAPDH expression. Bar graphs show the mean ± SEM; three MN spheroid batches per time point (14 and 28 days). Each MN spheroid batch was obtained from iPSC-derived MNPC batches at passage 3 generated through independent differentiation processes. Significance was determined for each gene using a one-way ANOVA. Next, post Dunnett’s tests using iPSCs (NANOG and OCT) or MNPCs (PAX6, OLIG2, HB9, ISL1, CHAT, MAP2, VSX2, SIM1, and MBP) as reference samples were performed. * *p* ≤ 0.05; ** *p* ≤ 0.01; *** *p* ≤ 0.001; **** *p* ≤ 0.0001.

**Figure 4:**
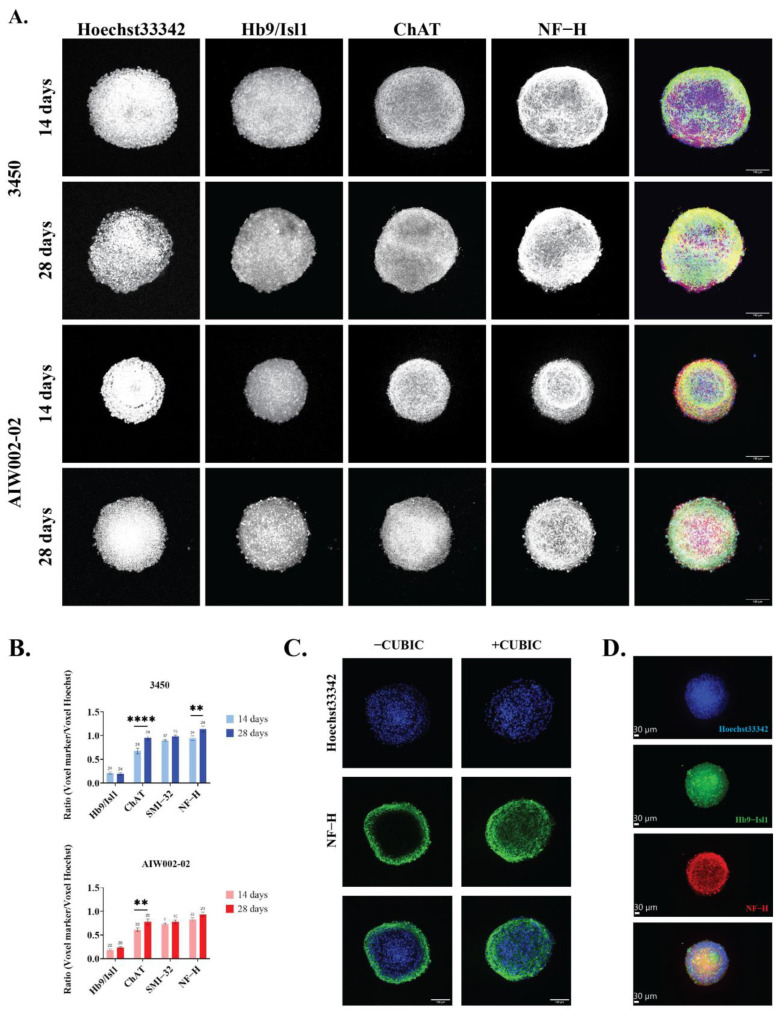
**Image profiling of iPSC-derived MN spheroids.** (**A**) Characterization of MN spheroids by immunostaining showed that MN spheroids generated from the 3450 and AIW002-02 iPSC lines express the specific MN markers Hb9, Isl1, CHAT, and SMI−32 (not shown), as well as the neuronal marker NF−H. Merge shows overlay of Hb9/Isl1 (blue), CHAT (red), and NF−H (green). (**B**) The presence of each MN marker was quantified using an in-house MATLAB pipeline. Graph bars show the mean ± SEM; for each cell line, each batch of three batches of iPSC-derived MNPCs generated through independent differentiation processes were used to generate two MN spheroid batches. A minimum of 9 MN spheroids were required for quantification, and we ensured that at least 3 spheroids per MNPC batch were stained for each marker per cell line at each time point. The significance between the two different time points (14 days and 28 days) for each MN marker was determined by an unpaired *t*-student ** *p* < 0.01; **** *p* < 0.0001. (**C**) CUBIC, a tissue-clearing protocol, was developed to increase antibody penetrance and decrease optical opacity to perform the quantification of the different markers in a more precise manner. For each condition, a single image from the center of the Z-stack was selected to be presented. (**D**) Representative image of a MN spheroid reconstructed as a 3D structure using the IMARIS software from Oxford Instruments.

Next, we assessed the expression of MN markers in the MN spheroids derived from the two control lines (AIW002-02 and 3450) at the transcript and protein levels by qPCR (Figure 3) and immunofluorescent staining (Figure 4), respectively. For both 3450 (Figure 3A) and AIW002-02 (Figure 3B) lines, the expression of different genes at the transcript level was assessed at the iPSC (NANOG and OCT4), MNPC (OLIG2 and PAX6), and MN spheroid (HB9, ISL1, CHAT, and MAP2) stage. The expression levels were compared using a one-way ANOVA with a post Dunnett’s test in which the control condition has been defined based on the mRNA transcript assessed. The complete results of the statistical analyses can be found in Supplementary Table S3. As expected, the pluripotency markers NANOG and OCT4 were found to be downregulated at the MNPC and MN spheroid stages compared to the iPSC stage. Significantly higher expression levels of the MNPC markers PAX6 and OLIG2 were found upregulated at the MNPC stage compared to the iPSC and MN spheroid stages. Finally, the expression level of the neural marker MAP2, as well as the expression levels of the MN markers HB9, ISL1, and CHAT were found to be upregulated at the MN spheroid stage, while being almost absent at the iPSC and MNPC stages. Additionally, we assessed the expression levels of interneuron (VSX2 and SIM1), oligodendrocyte (MBP), and astrocyte (GFAP) markers to address the presence of other cell types within the MN spheroids. For both cell lines, we confirmed the upregulation of MN markers at the transcript level, indicating a successful differentiation towards MN identity. GFAP expression remained undetected at the different differentiation stages, suggesting the absence of astrocytes. However, we observed the expression of interneuron and oligodendrocyte markers, indicating the presence of these cell types within MN spheroids.

To determine the presence of the different markers at the protein level, we analyzed the images of the cleared immunostained MN spheroids at 14 and 28 days (Figure 4A) with our in-house MATLAB script. We confirmed the expression of the pan MN markers Hb9/Isl1 after 14 and 28 days of differentiation with no statistical difference observed at the different time points for either cell line (Figure 4B). The expression of CHAT, a marker associated with MN maturation, was also assessed, showing an increase of its expression at 28 days compared to 14 days for both cells lines (3450 = *p* < 0.0001; AIW002-02 = *p* < 0.01) (Figure 4B). The expression of neurofilament heavy (NF−H) subunit, a marker indicating neuronal identity, was also increased at 28 days in both cell lines, however, it only reached a statistical significance in one of the cells lines (3450 = *p* < 0.01) (Figure 4B). Finally, we assessed the expression of SMI−32, an antibody that recognizes the non-phosphorylated form of the neurofilament medium (NF−M) and NF−H subunits, which is known to be highly enriched in spinal motor neurons. The expression of SMI−32 was confirmed at the two time points for both cell lines. We observe a tendency of SMI−32 to increase its expression at 28 days; however, it did not reach the statistical significance for either of the cell lines (Figure 4B). MN spheroids were also stained for pluripotency (Oct3/4) and MNPC (Pax6 and Olig2) markers to confirm their downregulation (Supplementary Figure S4). The expression of Hb9, Isl1, CHAT, and NF−H confirmed that, at the protein level, the MN spheroids are composed primarily of neurons with an MN identity. Nevertheless, positive antibody staining for interneuron progenitor (NKX2.2), interneuron (Vsx2 and Uncx), and oligodendrocyte (O4 and MBP) markers (Supplementary Figure S4) confirms the presence of other cell types in the MN spheroids, as suggested by the qPCR analysis (Figure 3).

**Figure 5:**
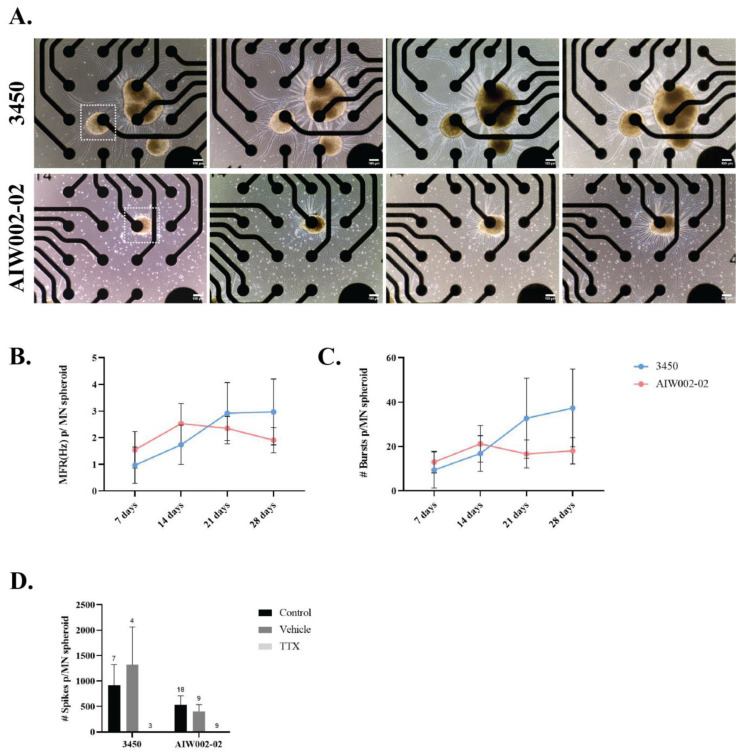
**Spontaneous firing activity of iPSC-derived MN spheroids at different time points.** (**A**) iPSC-derived MN spheroids were plated on wells of Cytoview 24-well MEA plates and recorded every 7 days until 28 days. Only MN spheroids placed at the center of an electrode (squares) through the entire recording time were considered for quantification. (**B**) Mean firing rate (MFR) and (**C**) number of bursts were plotted per individual MN spheroid. The connected scatter plots show the mean ± SEM; for each cell line, each batch of three batches of iPSC-derived MNPCs generated through independent differentiation processes were used to generate one MN spheroid batch. A minimum of nine MN spheroids were required for quantification to ensure that at least three spheroids per MNPC batch were quantified. (**D**) Tetrodotoxin (TTX) treatment blocked all firing in MN spheroids, ruling out system artifacts and confirming neural activity for both cell lines. H_2_O was used as vehicle. Graph bars show mean ± SEM.

Finally, we used the MEA system to assess the electrical activity of the MN spheroids. Only the spheroids placed at the center of the electrode were considered for quantification, meaning that each spheroid was in contact with a single electrode within the well (Figure 5A)**.** Action potentials (“spikes”) and groups of action potentials (“bursts”) were detected for the MN spheroids derived from the two control cell lines (3450 and AIW002-02) at different time points (7, 14, 21, and 28 days) (Supplementary Table S2). The mean firing rate (Hz), a ratio of the total number of spikes recorded over the duration of the recording, indicates that MN spheroids remain electrically active over time, as previously described for 2D monolayer iPSC-derived MN MEA recordings [60] (Figure 5B). Additionally, MN spheroids display burst firing over time (Figure 5C). Finally, treatment with TTX, a sodium channel blocker that inhibits the firing of action potential in neurons, confirmed the electrical activity of the MN spheroids, ruling out any potential artifacts coming from the system. Activity was not altered when a well was treated with vehicle (Figure 5D).

All graphs presented in the Results section were generated using GraphPad Prism (created by Dotmatics, version 9.1.1, Boston, MA, USA), www.graphpad.com, accessed 1 August 2022.

## 4. Discussion

Taken together, the results presented here demonstrate that we were able to develop a workflow to generate and characterize MN spheroids from iPSCs by modifying a protocol originally optimized to generate a 2D MN monolayer culture. A technical advantage associated with this protocol for the generation of iPSC-derived MNs is that it allows for the cryopreservation and expansion of MNPCs, making it time efficient. Importantly, we showed that thawed MNPCs used at different passages can produce MN spheroids in a reproducible manner and that culturing the cells in a 3D context did not interfere with the expression of MN identity markers, as shown by qPCR and immunofluorescent stainings. Remarkably, another technical advantage to growing the cells as 3D spheroids is that they can be grown and maintained for up to 28 days, and longer if needed, unlike 2D cultures that are often prone to clustering and detachment around 28 days in culture or earlier [31]. This becomes relevant, as we see a significant upregulation of CHAT in 28-day MN spheroids for both cell lines, which implies an increased maturation of the MNs over time, as shown by previous studies [61,62]. Interestingly, we observed different levels of significance between cell lines for some of the analyzed markers at both the gene expression and protein levels, which could be allusive to iPSC heterogeneity, a common obstacle in iPSC-based models [63]. The latter highlights the importance of using isogenic controls when referring to iPSC disease modelling instead of lines derived from healthy individuals when there is a monogenic cause of disease.

Previous studies have described the generation of ESC- and iPSC-derived MN spheroids using 96-well plates that favor the aggregation of cells by gravity. Characterization of these MN spheroids by means of qPCR has demonstrated the expression of progenitor (i.e., *OLIG2*, *NGN2*, *NeuroD1*), oligodendrocyte (i.e., *CLDN11*, *SOX10*, *OSP*), and astrocyte markers (i.e., GFAP) in aged spheroids [25]. However, except for GFAP and O4, there is a lack of characterization of these markers at the protein level [25,37,39,43]. Additionally, these studies bypass the analysis of interneuron markers, which share a common progenitor pool with MNs and are known to modulate MN synaptic activity [16,59]. Thus, this workflow provides a broader characterization of different markers not only at the expression level but also at the protein level. In fact, we found the presence of interneuron and oligodendrocyte markers within the MN spheroids, which is consistent with observations made by single-cell RNA sequencing [64] when iPSC-derived MNs were cultured as a 2D monolayer following the original protocol [16]. Therefore, this needs to be taken into consideration when assays are performed in these 3D models to avoid misinterpretations of results. Interestingly, opposed to similar work [25,37,39], we do not detect the expression of GFAP within our MN spheroids, raising the question as to whether an MNPC patterning step [43] is able to restrict more effectively the fate of the cells, giving rise only to cell types sharing a closest progenitor pool with MNs.

Even in small 3D samples such as spheroids, imaging is challenging due to the intrinsic opacity of biological tissues. Additionally, immunofluorescent staining of 3D structures was demonstrated to be difficult due to a low antibody penetrance, which can lead to the underestimation of specific staining for a given marker. Tissue clearing techniques are useful to reduce the water content of a sample and to remove lipids harbored within its cell membranes to (1) facilitate antibody penetrance and immunostaining and (2) reduce microscope light scattering to improve 3D image acquisition [46,65]. Among these, CUBIC is a rapid and inexpensive water-based clearing protocol that does not rely on the use of toxic reagents [46,53,66,67]. Recently, the CUBIC method was applied to clear spheroids coming from breasts cancer cell lines [68], however, to our knowledge, it has yet to be applied towards the clearing and imaging of neuronal spheres of any kind. Here, we succeeded in adapting the CUBIC protocol to clear MN spheroids (Figure 4D; Supplementary Video S1). The performance of this technique improved the immunofluorescent staining and subsequent image acquisition of our samples, allowing their characterization at the protein level. In contrast, when the CUBIC protocol was not performed, antibody penetration was limited to the periphery of the spheroid, making its center perceived as a necrotic core (Figure 4C).

MEA recordings of the MN spheroids confirmed the presence of neuronal activity within these 3D structures, as demonstrated by their ability to fire action potentials as isolated spikes or in bursts. Burst pattern measurements obtained by performing MEA recordings have been used to assess longitudinal electrical changes in 2D monolayer cortical cultures [69], however, to our knowledge there are not equivalent studies using MN cultures. Thus, this assay opens the possibility of using an MEA approach to analyze differences in burst patterns longitudinally when MN spheroids are derived from control vs. patient iPSC lines. Nevertheless, considering the presence of other cell types within the MN spheroids, it would be important to identify the different types of synapses present in these 3D structures by applying ion channel-specific antagonists. From the technical perspective, placing the MN spheroids at the center of a single electrode is challenging. Therefore, for future functional characterization, we acknowledge that a better approach would be the use of high-density MEAs (HD-MEAs), which have been recently used to assess the electrical features of other 3D structures [70,71]. HD-MEAs are composed of thousands of electrodes with minimal space between them. Therefore, HD-MEA systems not only provide an increase of spatiotemporal resolution but they would also facilitate the seeding process of 3D structures. Another alternative is the dissociation of spheroids into a single cell suspension to obtain MEA recordings of a 2D monolayer [55], however, this can raise concerns about the identity of the surviving population of cells after the dissociation process.

Even though there has been an increase in studies making use of MN spheroids, this is the first time a broader characterization has been made for such 3D structures with tools specifically developed with potential for large-scale studies. This is relevant because iPSC-derived spheroids/neurospheres have proven to be easy to generate 3D models that can be used for screening purposes [72], an important step towards drug discovery for MNDs. Moreover, this model can be used as a cellular jigsaw, in which other iPSC-derived cell types can be added to form more advanced co-culture spheroids, opening the possibility to study non-cell autonomous manifestation of the diseases.

## Figures and Tables

**Table 1:** **List of media and biochemicals**.

Reagents	Supplier/Manufacturer	Working Concentration	Catalogue Number
Accutase *	Thermo Fisher Scientific, Waltham, MA, USA	1X	A1110501
Antibiotic-Antimycotic (Anti-Anti)	Thermo Fisher Scientific, Waltham, MA, USA	1X	15240-062
B-27 supplement *	Thermo Fisher Scientific, Waltham, MA, USA	0.5X	17504-044
BDNF *	PeproTech, Cranbury, NJ, USA	10 ng/mL	450-02
CHIR-99021	Selleckchem, Houston, TX, USA	3 μM or 1 μM	S2924
Compound E	STEMCELL Technologies, Vancouver, BC, Canada	0.1 μM	73954
Dimethyl sulfoxide (DMSO)	Thermo Fisher Scientific, Waltham, MA, USA	10%	BP231-1
DMEM/F-12 medium	Thermo Fisher Scientific, Waltham, MA, USA	1X	10565018
DMH1	Selleckchem, Houston, TX, USA	2 μM	S7146
E8 medium	Thermo Fisher Scientific, Waltham, MA, USA	1X	A1517001
Fetal bovine serum (FBS)	Thermo Fisher Scientific, Waltham, MA, USA	1X	12484-028
Gentle cell dissociation reagent (GCDR)	STEMCELL Technologies, Vancouver, BC, Canada	1X	07174
GlutaMAX supplement	Thermo Fisher Scientific, Waltham, MA, USA	0.5X	35050-061
Laminin *	Sigma-Aldrich, St. Louis, MO, USA	5 μg/mL	L2020
L-Ascorbic acid (AA)	Sigma-Aldrich, St. Louis, MO, USA	100 μM	A5960
Matrigel *	Corning, New York, NY, USA	1X	354277
MTeSR1 medium	STEM CELL Technologies, Vancouver, BC, Canada	1X	85850
N-2 supplement *	Thermo Fisher Scientific, Waltham, MA, USA	0.5X	17502-048
Neurobasal medium	Thermo Fisher Scientific, Waltham, MA, USA	1X	21103-049
Phosphate buffer saline (PBS)	Wisent Products, Saint-Jean-Baptiste, QC, Canada	1X	311-010-CL
Poly-L-ornithine (PLO)	Sigma-Aldrich, St. Louis, MO, USA	10 μg/mL	P3655
Purmorphamine (Pur)	Sigma-Aldrich, St. Louis, MO, USA	0.5 μM or 0.1 μM	SML-0868
Retinoic acid (RA)	Sigma-Aldrich, St. Louis, MO, USA	0.1 μM or 0.5 μM	R2625
SB431542	Selleckchem, Houston, TX, USA	2 μM	S1067
Valproic acid (VPA)	Sigma-Aldrich, St. Louis, MO, USA	0.5 μM	P4543
Y-27632 (ROCK inhibitor)	Selleckchem, Houston, TX, USA	10 μM	S1049

* Note: Biochemicals with an asterisk are more susceptible to lot-to-lot variability. The main reason is the production source, either animal or human. It is therefore important to keep track of lot numbers. Regarding the Accutase solution, we noticed lot-to-lot variability in enzyme efficiency, as previously reported [46]. To compensate for weaker enzyme activity, incubate for a longer time until the cells detach properly.

**Table 2:** **Media composition**.

Media	Components
Neural induction medium	1:1 Neurobasal: DMEM/F12
	1X Anti-Anti
	0.5X N-2
	0.5X B-27
	0.5X GlutaMAX supplement
	100 μM AA
	3 μM CHIR-99021
	2 μM SB431542
	2 μM DMH1
MNPC patterning medium	1:1 Neurobasal: DMEM/F12
	1X Anti-Anti
	0.5X N-2
	0.5X B-27
	0.5X GlutaMAX supplement
	100 μM AA
	1 μM CHIR-99021
	2 μM DMH1
	2 μM SB431542
	0.1 μM RA
	0.5 μM Pur
MNPC expansion medium	1:1 Neurobasal: DMEM/F12
	1X Anti-Anti
	0.5X N-2
	0.5X B-27
	0.5X GlutaMAX supplement
	100 μM AA
	3 μM CHIR-99021
	2 μM DMH1
	2 μM SB431542
	0.1 μM RA
	0.5 μM Pur
	0.5 μM VPA
MN induction and maturation medium *	1:1 Neurobasal: DMEM/F12
	1X Anti-Anti
	0.5X N-2
	0.5X B-27
	0.5X GlutaMAX supplement
	100 μM AA
	0.5 μM RA
	0.1 μM Pur
	0.1 μM Compound E
	10 ng/mL BDNF

* The MN induction and maturation medium composition used in this study lacks two of the growth factors (CNTF and IGF-1) found in the original protocol [16]. This decision was made to avoid technical challenges in future experiments, as (a) skeletal muscle, a cell type often co-cultured with MNs, undergoes hypertrophy in the presence of IGF-1 [47] and (b) a disease phenotype could be masked in MN spheroids generated from patient iPSC lines grown in the presence of CNTF, which promotes neurite formation and outgrowth [48].

**Table 3:** **List of reagents**.

Reagents	Supplier/Manufacturer	Final Working Concentration per Reaction Tube	Volume (µL)	Catalogue Number
2X no UNG Taqman ^TM^ fast advanced master mix	Thermo Fisher Scientific, Waltham, MA, USA	N/A	N/A	A44360
miRNeasy micro kit	Qiagen, Hilden, Germany	N/A	N/A	217004
**M1 mix**				
Distilled water	N/A	N/A	12 µl	N/A
dNTPs	New England BioLabs, Whitby, ON, Canada	0.5 Mm	2 µL of a 10 Mm stock	N0447L
Random primers	Thermo Fisher Scientific, Waltham, MA, USA	12.5 ng/Μl	2 µL of a 250 ng/µL stock	48190011
**M2 mix**				
DTT	Thermo Fisher Scientific, Waltham, MA, USA	0.01 M	4 µL of 0.1 M stock	28025013
First strand buffer	Thermo Fisher Scientific, Waltham, MA, USA	1X	8 µL of 5X stock	28025013
M-MLV RT	Thermo Fisher Scientific, Waltham, MA, USA	400 U	2 µL of 200 U/µL M-MLV RT	28025013

**Table 4:** **Probes/primers**.

Gene Target	Reference	Supplier
ACTβ	Hs01060665_g1	Thermo Fisher Scientific
ChAT	Hs00758143_m1	Thermo Fisher Scientific
GAPDH	Hs02786624_g1	Thermo Fisher Scientific
GFAP	Hs00909233_m1	Thermo Fisher Scientific
HB9	Hs00907365_m1	Thermo Fisher Scientific
ISL1	Hs00158126_m1	Thermo Fisher Scientific
MAP2	Hs00258900_m1	Thermo Fisher Scientific
MBP	Hs00921945_m1	Thermo Fisher Scientific
NANOG	Hs02387400_g1	Thermo Fisher Scientific
OCT4	Hs04260367_gH	Thermo Fisher Scientific
OLIG2	Hs00377820_m1	Thermo Fisher Scientific
PAX6	Hs01088114_m1	Thermo Fisher Scientific
SIM1	Hs00231914_m1	Thermo Fisher Scientific
VSX2	Hs01584046_m1	Thermo Fisher Scientific

**Table 5:** **List of reagents**.

Reagents	Supplier/Manufacturer	Working Concentration	Catalogue Number
16% formaldehyde (FA)	Thermo Fisher Scientific, Waltham, MA, USA	4%	28,908
Hoechst33342	Thermo Fisher Scientific, Waltham, MA, USA	1:1000	H3570
Phosphate buffer saline (PBS)	Wisent Bioproducts, Saint-Jean-Baptiste, QC, Canada	1X	311-010-CL

**Table 6:** **CUBIC reagent 1 (R1) and CUBIC reagent 2 (R2) compositions**.

Reagents	Supplier/Manufacturer	Working Concentration	Catalogue Number
**R1**			
dH_2_O	N/A	35% by wt	N/A
Urea	Sigma-Aldrich, St. Louis, MO, USA	25% by wt	15604
Quadrol (N, N, N’, N’-tetrakis (2-hydroxy-propyl) ethylenediamine)	Sigma-Aldrich, St. Louis, MO, USA	25% by wt	122262
Triton X-100	Bioshop, Burlington, ON, Canada	15% by wt	TRX506
**R2**			
dH_2_O	N/A	15% by wt	N/A
Triethanolamine	Thermo Fisher Scientific, Waltham, MA, USA	10% by wt	T407-500
D-sucrose	Thermo Fisher Scientific, Waltham, MA, USA	50% by wt	BP220-1
Urea	Sigma-Aldrich, St. Louis, MO, USA	25% by wt	15604

**Table 7:** **Blocking solution composition**.

Reagents	Supplier/Manufacturer	Working Concentration	Catalogue Number
Bovine serum albumin (BSA)	Wisent Bioproducts, Saint-Jean-Baptiste, QC, Canada	0.05%	800-095-CG
Normal donkey serum (NDS)	Millipore, Burlington, MA, USA	5%	S30
Phosphate buffer saline (PBS)	Wisent Bioproducts, Saint-Jean-Baptiste, QC, Canada	1X	311-010-CL
Triton X-100	Bioshop, Burlington, ON, Canada	0.2%	TRX506

**Table 8:** **Primary and secondary antibodies**.

Primary Antibodies			
Antibody	Host Species	Working Dilution	Reference
ChAT	Goat	1:100	Millipore; Cat. No. MAB144P
Hb9 (81.5C10)	Mouse	1:50	DSHB
Isl1 (40.2D6)	Mouse	1:50	DSHB
Ki67	Mouse	1:200	BD Biosciences; Cat. No. 556003
MBP	Rat	1:200	Novusbio; Cat. No. NB600-717
Nestin	Mouse	1:250	Abcam; Cat. No. ab92391
Neurofilament-H	Chicken	1:1000	Abcam; Cat. No. ab4680
Nkx2.2	Mouse	1:100	DSHB
Nkx6.1	Mouse	1:100	DSHB
O4 *	Mouse	1:200	R&D System; Cat. No. MAB1326
Oct3/4	Goat	1:500	Santa Cruz; Cat. No. sc-8628
Olig2	Rabbit	1:100	Millipore; Cat No. AB 9610
Pax6	Mouse	1:100	DSHB
SMI-32	Mouse	1:100	Biolegend; Cat. No. 801701
Sox1	Goat	1:100	R&D System; Cat. No. AF3369
Uncx	Rabbit	1:250	Novusbio; Cat. No. NBP2-56480
Vsx2 (Chx10)	Mouse	1:250	Santa-Cruz; Cat. No. sc-365519
**Secondary Antibodies**			
**Host Species**	**Target Species—Fluorophore**	**Working Dilution**	**Reference**
Donkey	Goat IgG-DyLight 550	1:250	Abcam; Cat No. ab96932
Donkey	Goat IgG-AlexaFluor 647	1:250	Jackson Immunoresearch; Cat. No. 703-605-155
Donkey	Mouse IgG-DyLight 488	1:250	Abcam; Cat. No. ab96875
Donkey	Chicken IgG-AlexaFluor 647	1:250	Jackson Immunoresearch; Cat. No. 703-605-155
Donkey	Rabbit IgG-DyLight 550	1:250	Abcam; Cat. No. ab96892
Donkey	Rabbit-DyLight 488	1:250	Abcam; Cat. No. ab96891
Goat	Rat IgG-DyLight 488	1:250	Abcam; Cat. No. ab96887

* Note: To obtain optimal results with O4 primary antibody, perform a live staining for 30 min at 37 °C before formaldehyde fixation.

**Table 9:** **List of reagents**.

Reagents	Supplier/Manufacturer	Working Concentration	Catalogue Number
Laminin	Invitrogen, Waltham, MA, USA	5 μg/mL	23017015
Poly-L-ornithine (PLO)	Sigma-Aldrich, St. Louis, MO, USA	10 μg/mL	P3655
Tetrodotoxin (TTX)	Sigma-Aldrich, St. Louis, MO, USA	1 mM	T8024

**Table 10:** **Composition of aCSF**.

Reagents **	Supplier/Manufacturer	Working Concentration	Catalogue Number
CaCl_2_	Thermo Fisher Scientific, Waltham, MA, USA	1.6 mM	AC423525000
D-glucose *	Sigma-Aldrich, St. Louis, MO, USA	5.5 mM	G8270
KCl	Bioshop, Burlington, ON, Canada	4 mM	POC888.5
KH_2_PO_4_	Thermo Fisher Scientific, Waltham, MA, USA	1.18 mM	AC424200250
MgSO_4_	Thermo Fisher Scientific, Waltham, MA, USA	1.17 mM	AC423905000
NaCl	BioShop, Burlington, ON, Canada	119 mM	SOD004.5
NaHCO_3_ *	Thermo Fisher Scientific, Waltham, MA, USA	24 mM	AC424270010

* These reagents are not added to the 10X stock solution to prevent the growth of microorganisms. They are added to the 1X solution before every recording experiment. ** Add 2 g of NaCl to 10X solution stock for adjusting osmolality 305 mOsM.

## Data Availability

The data presented in this study are available on request from the corresponding author.

## Supplementary Materials

The following supporting information can be downloaded at: https://www.mdpi.com/article/10.3390/cells12040545/s1, Figure S1: Motor neuron neural progenitor cell (MNPC) characterization by immunofluorescent staining; Figure S2: Inappropriate formation of MN spheroids; Figure S3: CellProfiler macro to perform the size profiling of the MN spheroids; Figure S4: Image profiling of iPSC-derived MN spheroids to identify different cell types; Figure S5: Graphic summary for the generation and characterizacion of iPSC-derived MN spheroids; Table S1: List of consumables and equipment; Table S2: Raw MEA data; Table S3: qPCR statistics; Video S1: Rotating 3D reconstruction of MN spheroid.

## References

[1] Sanes J.R., Lichtman J.W. (1999). Development of the vertebrate neuromuscular junction. Annu. Rev. Neurosci..

[2] Tiryaki E., Horak H.A. (2014). ALS and other motor neuron diseases. Continuum.

[3] Park J., Kim J.E., Song T.J. (2022). The Global Burden of Motor Neuron Disease: An Analysis of the 2019 Global Burden of Disease Study. Front. Neurol..

[4] Gois A.M., Mendonça D.M.F., Freire M.A.M., Santos J.R. (2020). In vitro and in vivo models of amyotrophic lateral sclerosis: An updated overview. Brain Res. Bull..

[5] Slanzi A., Iannoto G., Rossi B., Zenaro E., Constantin G. (2020). In vitro Models of Neurodegenerative Diseases. Front. Cell Dev. Biol..

[6] Sances S., Bruijn L.I., Chandran S., Eggan K., Ho R., Klim J.R., Livesey M.R., Lowry E., Macklis J.D., Rushton D. (2016). Modeling ALS with motor neurons derived from human induced pluripotent stem cells. Nat. Neurosci..

[7] Takahashi K., Yamanaka S. (2006). Induction of pluripotent stem cells from mouse embryonic and adult fibroblast cultures by defined factors. Cell.

[8] Takahashi K., Tanabe K., Ohnuki M., Narita M., Ichisaka T., Tomoda K., Yamanaka S. (2007). Induction of pluripotent stem cells from adult human fibroblasts by defined factors. Cell.

[9] Fujimori K., Ishikawa M., Otomo A., Atsuta N., Nakamura R., Akiyama T., Hadano S., Aoki M., Saya H., Sobue G. (2018). Modeling sporadic ALS in iPSC-derived motor neurons identifies a potential therapeutic agent. Nat. Med..

[10] Ratti A., Gumina V., Lenzi P., Bossolasco P., Fulceri F., Volpe C., Bardelli D., Pregnolato F., Maraschi A., Fornai F. (2020). Chronic stress induces formation of stress granules and pathological TDP-43 aggregates in human ALS fibroblasts and iPSC-motoneurons. Neurobiol. Dis..

[11] Kim B.W., Ryu J., Jeong Y.E., Kim J., Martin L.J. (2020). Human motor neurons with SOD1-G93A mutation generated from CRISPR/Cas9 gene-edited iPSCs develop pathological features of amyotrophic lateral sclerosis. Front. Cell Neurosci..

[12] Deneault E., Chaineau M., Nicouleau M., Castellanos Montiel M.J., Franco Flores A.K., Haghi G., Chen C.X., Abdian N., Shlaifer I., Beitel L.K. (2022). A streamlined CRISPR workflow to introduce mutations and generate isogenic iPSCs for modeling amyotrophic lateral sclerosis. Methods.

[13] Selvaraj B.T., Livesey M.R., Zhao C., Gregory J.M., James O.T., Cleary E.M., Chouhan A.K., Gane A.B., Perkins E.M., Dando O. (2018). C9ORF72 repeat expansion causes vulnerability of motor neurons to Ca^2+^-permeable AMPA receptor-mediated excitotoxicity. Nat. Commun..

[14] Guo W., Naujock M., Fumagalli L., Vandoorne T., Baatsen P., Boon R., Ordovás L., Patel A., Welters M., Vanwelden T. (2017). HDAC6 inhibition reverses axonal transport defects in motor neurons derived from FUS-ALS patients. Nat. Commun..

[15] Boulting G.L., Kiskinis E., Croft G.F., Amoroso M.W., Oakley D.H., Wainger B.J., Williams D.J., Kahler D.J., Yamaki M., Davidow L. (2011). A functionally characterized test set of human induced pluripotent stem cells. Nat. Biotechnol..

[16] Du Z.W., Chen H., Liu H., Lu J., Qian K., Huang C.L., Zhong X., Fan F., Zhang S.C. (2015). Generation and expansion of highly pure motor neuron progenitors from human pluripotent stem cells. Nat. Commun..

[17] Qu Q., Li D., Louis K.R., Li X., Yang H., Sun Q., Crandall S.R., Tsang S., Zhou J., Cox C.L. (2014). High-efficiency motor neuron differentiation from human pluripotent stem cells and the function of Islet-1. Nat. Commun..

[18] Amoroso M.W., Croft G.F., Williams D.J., O’Keeffe S., Carrasco M.A., Davis A.R., Roybon L., Oakley D.H., Maniatis T., Henderson C.E. (2013). Accelerated high-yield generation of limb-innervating motor neurons from human stem cells. J. Neurosci..

[19] Dimos J.T., Rodolfa K.T., Niakan K.K., Weisenthal L.M., Mitsumoto H., Chung W., Croft G.F., Saphier G., Leibel R., Goland R. (2008). Induced pluripotent stem cells generated from patients with ALS can be differentiated into motor neurons. Science.

[20] Maury Y., Côme J., Piskorowski R.A., Salah-Mohellibi N., Chevaleyre V., Peschanski M., Martinat C., Nedelec S. (2015). Combinatorial analysis of developmental cues efficiently converts human pluripotent stem cells into multiple neuronal subtypes. Nat. Biotechnol..

[21] Karpe Y., Chen Z., Li X.J. (2021). Stem cell models and gene targeting for human motor neuron diseases. Pharmaceuticals.

[22] Guo W., Fumagalli L., Prior R., Van Den Bosch L. (2017). Current advances and limitations in modeling ALS/FTD in a dish using induced pluripotent stem cells. Front. Neurosci..

[23] Afshar Bakooshli M., Lippmann E.S., Mulcahy B., Iyer N., Nguyen C.T., Tung K., Stewart B.A., van den Dorpel H., Fuehrmann T., Shoichet M. (2019). A 3D culture model of innervated human skeletal muscle enables studies of the adult neuromuscular junction. eLife.

[24] De Leeuw S.M., Davaz S., Wanner D., Milleret V., Ehrbar M., Gietl A., Tackenberg C. (2021). Increased maturation of iPSC-derived neurons in a hydrogel-based 3D culture. J. Neurosci. Methods.

[25] Osaki T., Uzel S.G.M., Kamm R.D. (2018). Microphysiological 3D model of amyotrophic lateral sclerosis (ALS) from human iPS-derived muscle cells and optogenetic motor neurons. Sci. Adv..

[26] Brännvall K., Bergman K., Wallenquist U., Svahn S., Bowden T., Hilborn J., Forsberg-Nilsson K. (2007). Enhanced neuronal differentiation in a three-dimensional collagen-hyaluronan matrix. J. Neurosci. Res..

[27] Smith I., Haag M., Ugbode C., Tams D., Rattray M., Przyborski S., Bithell A., Whalley B.J. (2015). Neuronal-glial populations form functional networks in a biocompatible 3D scaffold. Neurosci. Lett..

[28] Vagaska B., Gillham O., Ferretti P. (2020). Modelling human CNS injury with human neural stem cells in 2- and 3-Dimensional cultures. Sci. Rep..

[29] Thiry L., Clément J.P., Haag R., Kennedy T.E., Stifani S. (2022). Optimization of long-term human iPSC-derived spinal motor neuron culture using a dendritic polyglycerol amine-based substrate. ASN Neuro.

[30] Taga A., Dastgheyb R., Habela C., Joseph J., Richard J.P., Gross S.K., Lauria G., Lee G., Haughey N., Maragakis N.J. (2019). Role of human-induced pluripotent stem cell-derived spinal cord astrocytes in the functional maturation of motor neurons in a multielectrode array system. Stem Cells Transl. Med..

[31] Milky B., Zabolocki M., Al-Bataineh S.A., van den Hurk M., Greenberg Z., Turner L., Mazzachi P., Williams A., Illeperuma I., Adams R. (2022). Long-term adherence of human brain cells in vitro is enhanced by charged amine-based plasma polymer coatings. Stem Cell Rep..

[32] Toli D., Buttigieg D., Blanchard S., Lemonnier T., Lamotte d’Incamps B., Bellouze S., Baillat G., Bohl D., Haase G. (2015). Modeling amyotrophic lateral sclerosis in pure human iPSc-derived motor neurons isolated by a novel FACS double selection technique. Neurobiol. Dis..

[33] Faustino Martins J.M., Fischer C., Urzi A., Vidal R., Kunz S., Ruffault P.L., Kabuss L., Hube I., Gazzerro E., Birchmeier C. (2020). Self-organizing 3D human trunk neuromuscular organoids. Cell Stem Cell.

[34] Pereira J.D., DuBreuil D.M., Devlin A.C., Held A., Sapir Y., Berezovski E., Hawrot J., Dorfman K., Chander V., Wainger B.J. (2021). Human sensorimotor organoids derived from healthy and amyotrophic lateral sclerosis stem cells form neuromuscular junctions. Nat. Commun..

[35] Hor J.H., Ng S.Y. (2020). Generating ventral spinal organoids from human induced pluripotent stem cells. Methods Cell Biol..

[36] Andersen J., Revah O., Miura Y., Thom N., Amin N.D., Kelley K.W., Singh M., Chen X., Thete M.V., Walczak E.M. (2020). Generation of functional human 3D cortico-motor assembloids. Cell.

[37] Osaki T., Uzel S.G.M., Kamm R.D. (2020). On-chip 3D neuromuscular model for drug screening and precision medicine in neuromuscular disease. Nat. Protoc..

[38] Machado C.B., Pluchon P., Harley P., Rigby M., Gonzalez Sabater V., Stevenson D.C., Hynes S., Lowe A., Burrone J., Viasnoff V. (2019). In vitro modelling of nerve-muscle connectivity in a compartmentalised tissue culture device. Adv. Biosyst..

[39] Kawada J., Kaneda S., Kirihara T., Maroof A., Levi T., Eggan K., Fujii T., Ikeuchi Y. (2017). Generation of a motor nerve organoid with human stem cell-derived neurons. Stem Cell Rep..

[40] Centeno E.G.Z., Cimarosti H., Bithell A. (2018). 2D versus 3D human induced pluripotent stem cell-derived cultures for neurodegenerative disease modelling. Mol. Neurodegener..

[41] Hughes C.S., Postovit L.M., Lajoie G.A. (2010). Matrigel: A complex protein mixture required for optimal growth of cell culture. Proteomics.

[42] Yamamoto K., Yamaoka N., Imaizumi Y., Nagashima T., Furutani T., Ito T., Okada Y., Honda H., Shimizu K. (2021). Development of a human neuromuscular tissue-on-a-chip model on a 24-well-plate-format compartmentalized microfluidic device. Lab Chip.

[43] Osaki T., Sivathanu V., Kamm R.D. (2018). Engineered 3D vascular and neuronal networks in a microfluidic platform. Sci. Rep..

[44] Chen X., Rocha C., Rao T., Durcan T.M. (2019). Motor neuron induction and differentiation (V2.0). Zenodo.

[45] Chen C.X., Abdian N., Maussion G., Thomas R.A., Demirova I., Cai E., Tabatabaei M., Beitel L.K., Karamchandani J., Fon E.A. (2021). A Multistep Workflow to Evaluate Newly Generated iPSCs and Their Ability to Generate Different Cell Types. Methods Protoc..

[46] Mohamed N.V., Lépine P., Lacalle-Aurioles M., Sirois J., Mathur M., Reintsch W., Beitel L.K., Fon E.A., Durcan T.M. (2022). Microfabricated disk technology: Rapid scale up in midbrain organoid generation. Methods.

[47] Yoshida T., Delafontaine P. (2020). Mechanisms of IGF-1-mediated regulation of skeletal muscle hypertrophy and atrophy. Cells.

[48] Oyesiku N.M., Wigston D.J. (1996). Ciliary neurotrophic factor stimulates neurite outgrowth from spinal cord neurons. J. Comp. Neurol..

[49] Jones T.R., Kang I.H., Wheeler D.B., Lindquist R.A., Papallo A., Sabatini D.M., Golland P., Carpenter A.E. (2008). CellProfiler Analyst: Data exploration and analysis software for complex image-based screens. BMC Bioinform..

[50] Maussion G., Moalic J.M., Simonneau M., Gorwood P., Ramoz N. (2019). Increased expression of BDNF mRNA in the frontal cortex of autistic patients. Behav. Brain Res..

[51] Bell S., Maussion G., Jefri M., Peng H., Theroux J.F., Silveira H., Soubannier V., Wu H., Hu P., Galat E. (2018). Disruption of GRIN2B Impairs Differentiation in Human Neurons. Stem Cell Rep..

[52] Maussion G., Thomas R.A., Demirova I., Gu G., Cai E., Chen C.X., Abdian N., Strauss T.J.P., Kelaï S., Nauleau-Javaudin A. (2021). Auto-qPCR; a python-based web app for automated and reproducible analysis of qPCR data. Sci. Rep..

[53] Gómez-Gaviro M.V., Balaban E., Bocancea D., Lorrio M.T., Pompeiano M., Desco M., Ripoll J., Vaquero J.J. (2017). Optimized CUBIC protocol for three-dimensional imaging of chicken embryos at single-cell resolution. Development.

[54] Mohamed N.V., Sirois J., Ramamurthy J., Mathur M., Lépine P., Deneault E., Maussion G., Nicouleau M., Chen C.X., Abdian N. (2021). Midbrain organoids with an SNCA gene triplication model key features of synucleinopathy. Brain Commun..

[55] Kahn-Krell A., Pretorius D., Guragain B., Lou X., Wei Y., Zhang J., Qiao A., Nakada Y., Kamp T.J., Ye L. (2022). A three-dimensional culture system for generating cardiac spheroids composed of cardiomyocytes, endothelial cells, smooth-muscle cells, and cardiac fibroblasts derived from human induced-pluripotent stem cells. Front. Bioeng. Biotechnol..

[56] Lendahl U., Zimmerman L.B., McKay R.D. (1990). CNS stem cells express a new class of intermediate filament protein. Cell.

[57] Neely M.D., Litt M.J., Tidball A.M., Li G.G., Aboud A.A., Hopkins C.R., Chamberlin R., Hong C.C., Ess K.C., Bowman A.B. (2012). DMH1, a highly selective small molecule BMP inhibitor promotes neurogenesis of hiPSCs: Comparison of PAX6 and SOX1 expression during neural induction. ACS Chem. Neurosci..

[58] Gerdes J., Lemke H., Baisch H., Wacker H.H., Schwab U., Stein H. (1984). Cell cycle analysis of a cell proliferation-associated human nuclear antigen defined by the monoclonal antibody Ki-67. J. Immunol..

[59] Ogura T., Sakaguchi H., Miyamoto S., Takahashi J. (2018). Three-dimensional induction of dorsal, intermediate and ventral spinal cord tissues from human pluripotent stem cells. Development.

[60] Ronchi S., Buccino A.P., Prack G., Kumar S.S., Schröter M., Fiscella M., Hierlemann A. (2021). Electrophysiological phenotype characterization of human iPSC-derived neuronal cell lines by means of high-density microelectrode arrays. Adv. Biol..

[61] Phelps P.E., Barber R.P., Houser C.R., Crawford G.D., Salvaterra P.M., Vaughn J.E. (1984). Postnatal development of neurons containing choline acetyltransferase in rat spinal cord: An immunocytochemical study. J. Comp. Neurol..

[62] Sepehrimanesh M., Ding B. (2020). Generation and optimization of highly pure motor neurons from human induced pluripotent stem cells via lentiviral delivery of transcription factors. Am. J. Physiol. Cell Physiol..

[63] Volpato V., Webber C. (2020). Addressing variability in iPSC-derived models of human disease: Guidelines to promote reproducibility. Dis. Model. Mech..

[64] Thiry L., Hamel R., Pluchino S., Durcan T., Stifani S. (2020). Characterization of human iPSC-derived spinal motor neurons by single-cell RNA sequencing. Neuroscience.

[65] Zhao J., Lai H.M., Qi Y., He D., Sun H. (2021). Current Status of Tissue Clearing and the Path Forward in Neuroscience. ACS Chem. Neurosci..

[66] Susaki E.A., Tainaka K., Perrin D., Kishino F., Tawara T., Watanabe T.M., Yokoyama C., Onoe H., Eguchi M., Yamaguchi S. (2014). Whole-brain imaging with single-cell resolution using chemical cocktails and computational analysis. Cell.

[67] Susaki E.A., Tainaka K., Perrin D., Yukinaga H., Kuno A., Ueda H.R. (2015). Advanced CUBIC protocols for whole-brain and whole-body clearing and imaging. Nat. Protoc..

[68] Diosdi A., Hirling D., Kovacs M., Toth T., Harmati M., Koos K., Buzas K., Piccinini F., Horvath P. (2021). Cell lines and clearing approaches: A single-cell level 3D light-sheet fluorescence microscopy dataset of multicellular spheroids. Data Brief.

[69] Wagenaar D.A., Pine J., Potter S.M. (2006). An extremely rich repertoire of bursting patterns during the development of cortical cultures. BMC Neurosci..

[70] Sharf T., van der Molen T., Glasauer S.M.K., Guzman E., Buccino A.P., Luna G., Cheng Z., Audouard M., Ranasinghe K.G., Kudo K. (2022). Functional neuronal circuitry and oscillatory dynamics in human brain organoids. Nat. Commun..

[71] Passaro A.P., Stice S.L. (2020). Electrophysiological analysis of brain organoids: Current approaches and advancements. Front. Neurosci..

[72] Kobolak J., Teglasi A., Bellak T., Janstova Z., Molnar K., Zana M., Bock I., Laszlo L., Dinnyes A. (2020). Human induced pluripotent stem cell-derived 3D-neurospheres are suitable for neurotoxicity screening. Cells.

