# Supplementary material for: An Optimized Workflow to Generate and Characterize iPSC-Derived Motor Neuron (MN) Spheroids
Source: Cells. 2023 Feb 8;12(4):545. doi: 10.3390/cells12040545 (PMC9954647; doi:10.3390/cells12040545)
Supplement: Supplementary file 1 [file cells-12-00545-s001.zip › Castellanos-Montiel_Supplementary Material_Submission.pdf]

Supplementary Materials for

An optimized workflow to generate and characterize iPSC-derived motor neuron (MN) spheroids

María José Castellanos-Montiel, Mathilde Chaineau, Anna Kristyna Franco-Flores, Ghazal Haghi, Dulce Carrillo-Valenzuela, Wolfgang E. Reintsch, Carol X-Q Chen, Thomas M. Durcan

Corresponding author e-mail: thomas.durcan@mcgill.ca (T.M.D)

This PDF file includes:

Supplementary Table S1

Supplementary Figures S1–S4

Supplementary Table S1. List of consumables and equipment

Consumables and equipment	Supplier	Catalogue number
**Generation of iPSC-derived MN spheroids**		
100 mm culture dishes	Corning	353003
96 round bottom ultra-low attachment plates	Corning	CLS7007
Cell culture incubator	Thermo Fisher Scientific	Steri-Cycle Model 370 Ref#20
Cell scraper	Corning	CLS3010
Centrifuge	Eppendorf	022626001
Centrifuge with adapter for culture well plates	Eppendorf	5810R
Conical tube, 15mL	Thermo Fisher Scientific	352097
Cryovials	Sarstedt	72.379
Luna-II™ automated cell counter	Logos biosystems	L40002
Plastic serological pipet, 10mL	Thermo Fisher Scientific	13-678-11E
Plastic serological pipet, 1mL	Thermo Fisher Scientific	13-678-11B
Plastic serological pipet, 5mL	Thermo Fisher Scientific	13-678-11D
T-25 flasks	Thermo Fisher Scientific	12-556-009
T-75 flasks	Thermo Fisher Scientific	12-556-010
**Cell profiler macro for size profiling of MN spheroids**		
EVOS XL Core	Thermo Fisher Scientific	AMEX1000
JuLI™ Stage	NanoEntek	NANOJS1000S
**qPCR analysis of MN spheroids**		
MicroAmp™ 8 tube strip (0.2 mL)	Thermo Fisher Scientific	N8010580
MicroAmp™ optical 384-well plate reaction plate	Thermo Fisher Scientific	4309849
MicroAmp™ optical 8-cap strips	Thermo Fisher Scientific	4323032
MicroAmp™ optical adhesive	Thermo Fisher Scientific	4311971
Nanodrop	Thermo Fisher Scientific	ND-ONE-W
QuantStudio 5	Thermo Fisher Scientific	A28140
SimpliAmp™ thermal cycler	Thermo Fisher Scientific	A24811
**Fixation, tissue clearing and immunofluorescent staining of MN spheroids**		
0.6 mL locking-lid microcentrifuge tubes	Thermo Fisher Scientific	02-681-273
Benchtop shaking incubator	Corning	LSE™ 6790
Black bottom 96 well plate	Corning	353219
Nutating mixer	VWR	82007-202
Opera Phenix High-Content Screening System	PerkinElmer	N/A
Sterile cell strainer 40 μM	Thermo Fisher Scientific	22363547
Wide orifice low binding tips	Labcon	1164-965-008-9
**Microelectrode array (MEA) recordings of MN spheroids**		
0.2 μm filters	Thermo Fisher Scientific	09-719C
30 mL Syringe	VWR	76290-386
50 mL conical tube	Progene	71-5000-B
Axion Maestro Edge	Axion Biosystems	N/A
Cytoview 24-well MEA plates	Axion Biosystems	M384-Tmea-24w
Wide orifice low binding tips	Labcon	1164-965-008-9

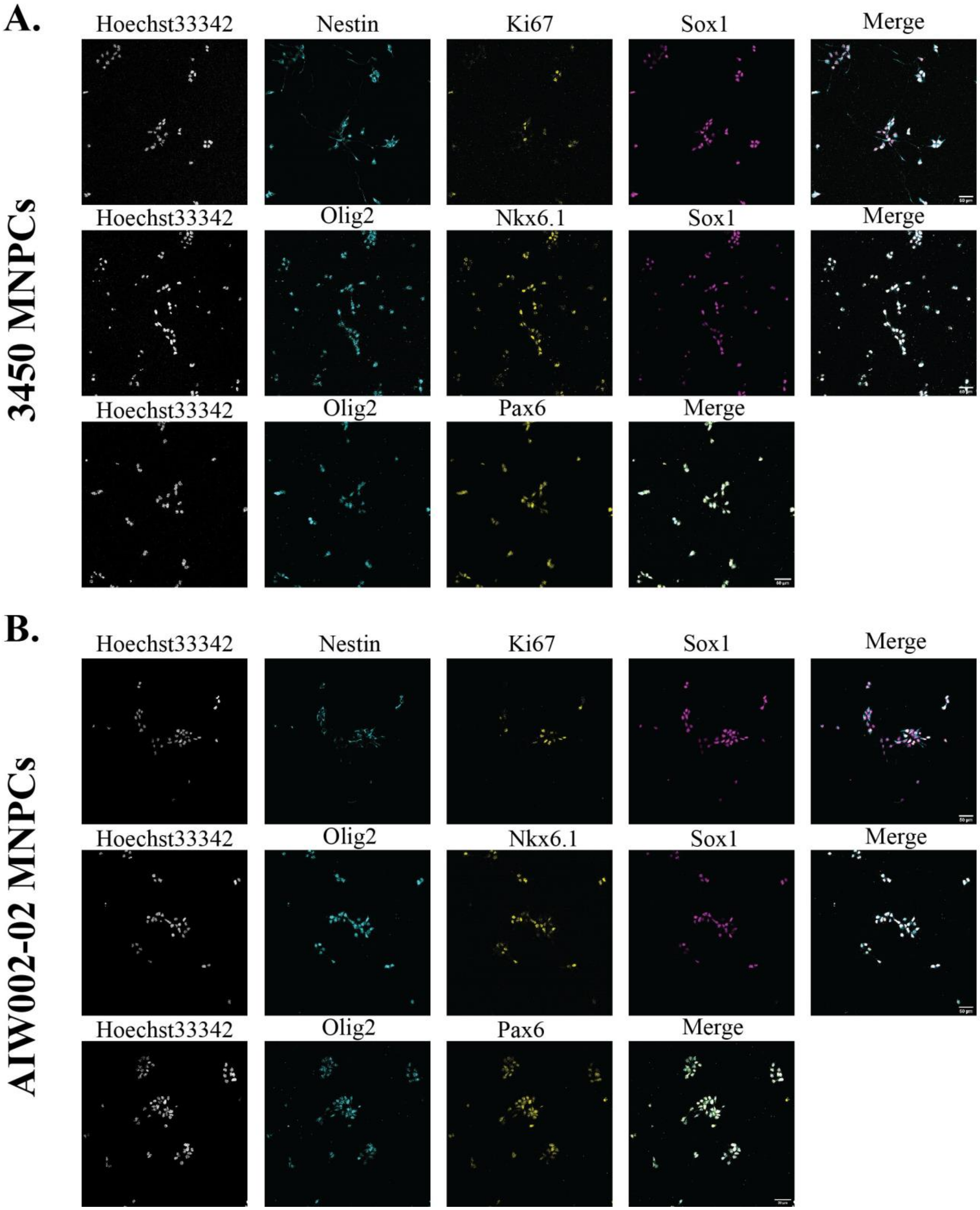

Supplementary Figure S1. Motor neuron neural progenitor cell (MNPC) characterization by immunofluorescent staining. A. 3450 and **B.** AIW002-02 MNPCs were positive for Sox1 and Nestin indicating their neural progenitor identity as well as Ki67, which demonstrates their proliferation capacity. Additionally, MNPCs are positive for the markers Pax6, Olig2 and NKx6.1 that indicate their successful specification towards motor neuron neural progenitor cells.

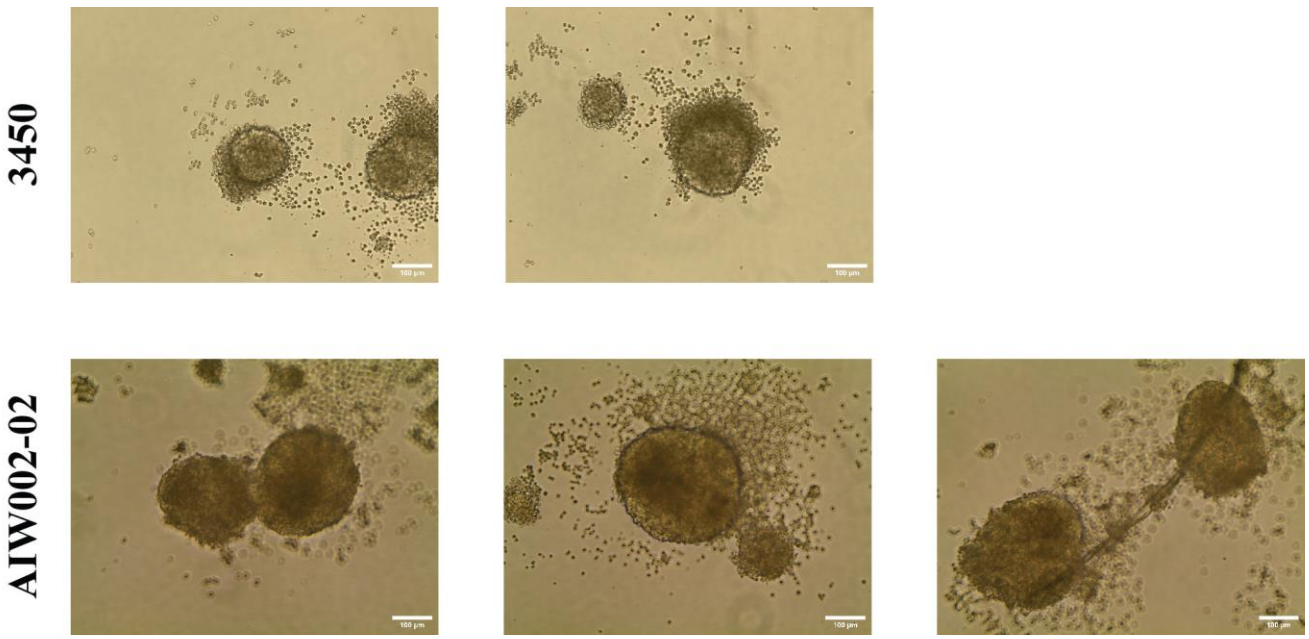

Supplementary Figure S2. Inappropriate formation of MN spheroids. Examples illustrating inappropriate formation of MN spheroids in random wells regardless of the iPSC line used.

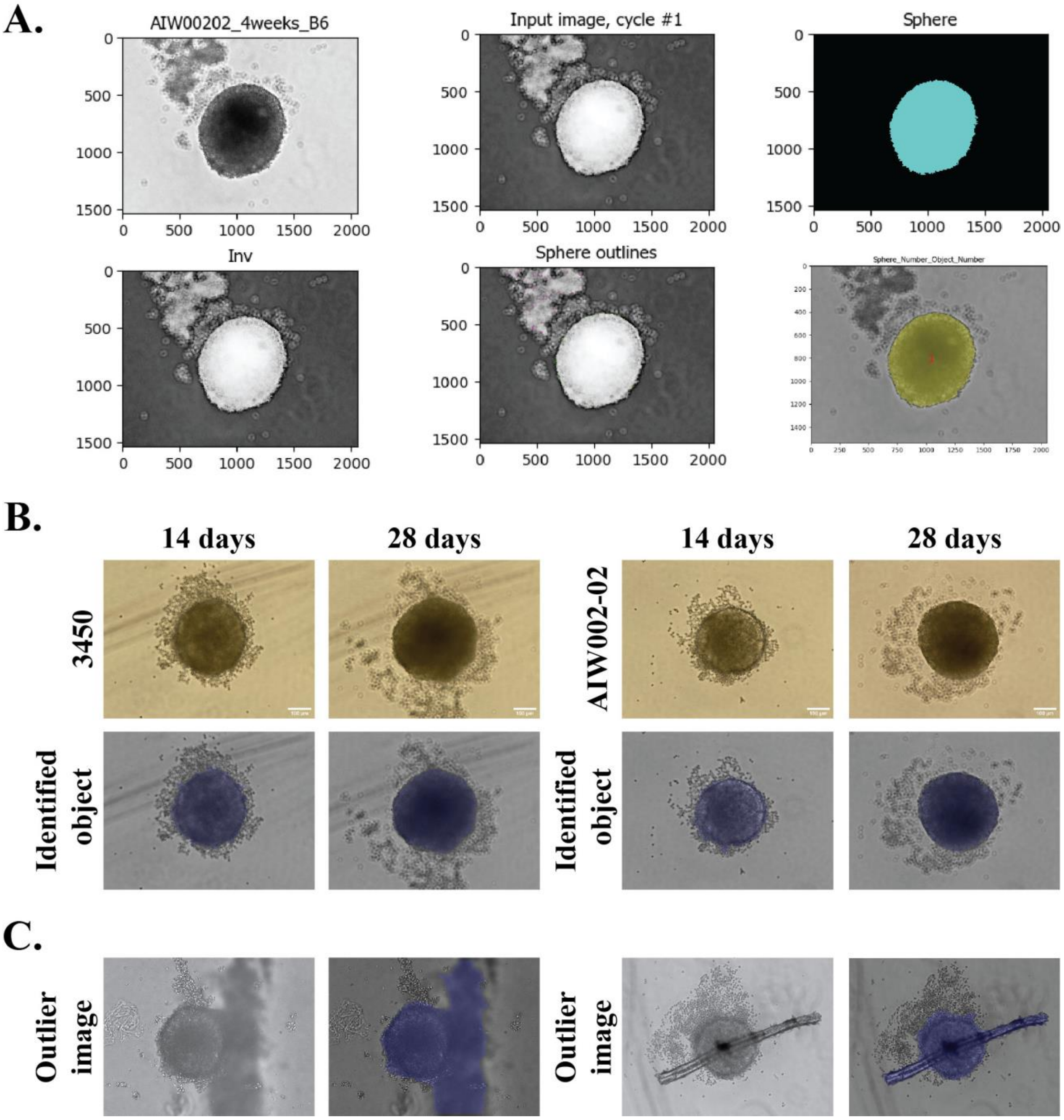

Supplementary Figure S3. CellProfiler macro to perform the size profiling of the MN spheroids. A) A data set of pictures taken with a bright-field microscope is inserted into a Cell Profiler pipeline. **B)** The pipeline processes the image to identify a primary object that is overlaid with the original image. **C)** Images in which the macro performed poorly are considered outliers and removed from the analyses.

A.

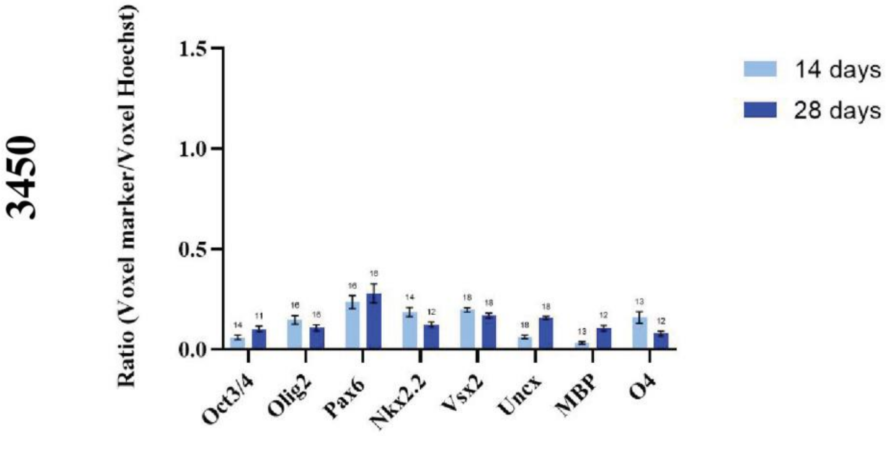

B.

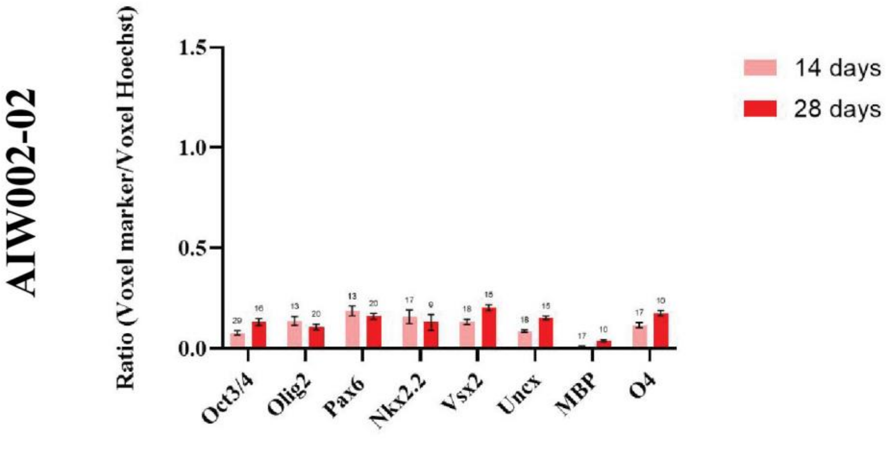

Supplementary Figure S4. Image profiling of iPSC-derived MN spheroids to identify different cell types. MN spheroids from **A)** 3450 and **B)** AIW002-02 control cell lines were stained for interneuron (Nkx2.2, Vsx2, Uncx) and oligodendrocyte markers (MBP, O4). The presence of these markers was quantified using an in-house MATLAB pipeline. Graph bars show the mean ± SEM; for each cell line, each batch of three batches of iPSC-derived MNPCs generated through independent differentiation processes was used to generate two MN spheroid batches. A minimum of 9 MN spheroids were required for quantification, and we ensured that at least 3 spheroids per MNPC batch where stained for each marker per cell line at each time point.
