# Supplementary figures and images for: An Optimized Workflow to Generate and Characterize iPSC-Derived Motor Neuron (MN) Spheroids
Source: Cells. 2023 Feb 8;12(4):545. doi: 10.3390/cells12040545 (PMC9954647; doi:10.3390/cells12040545)

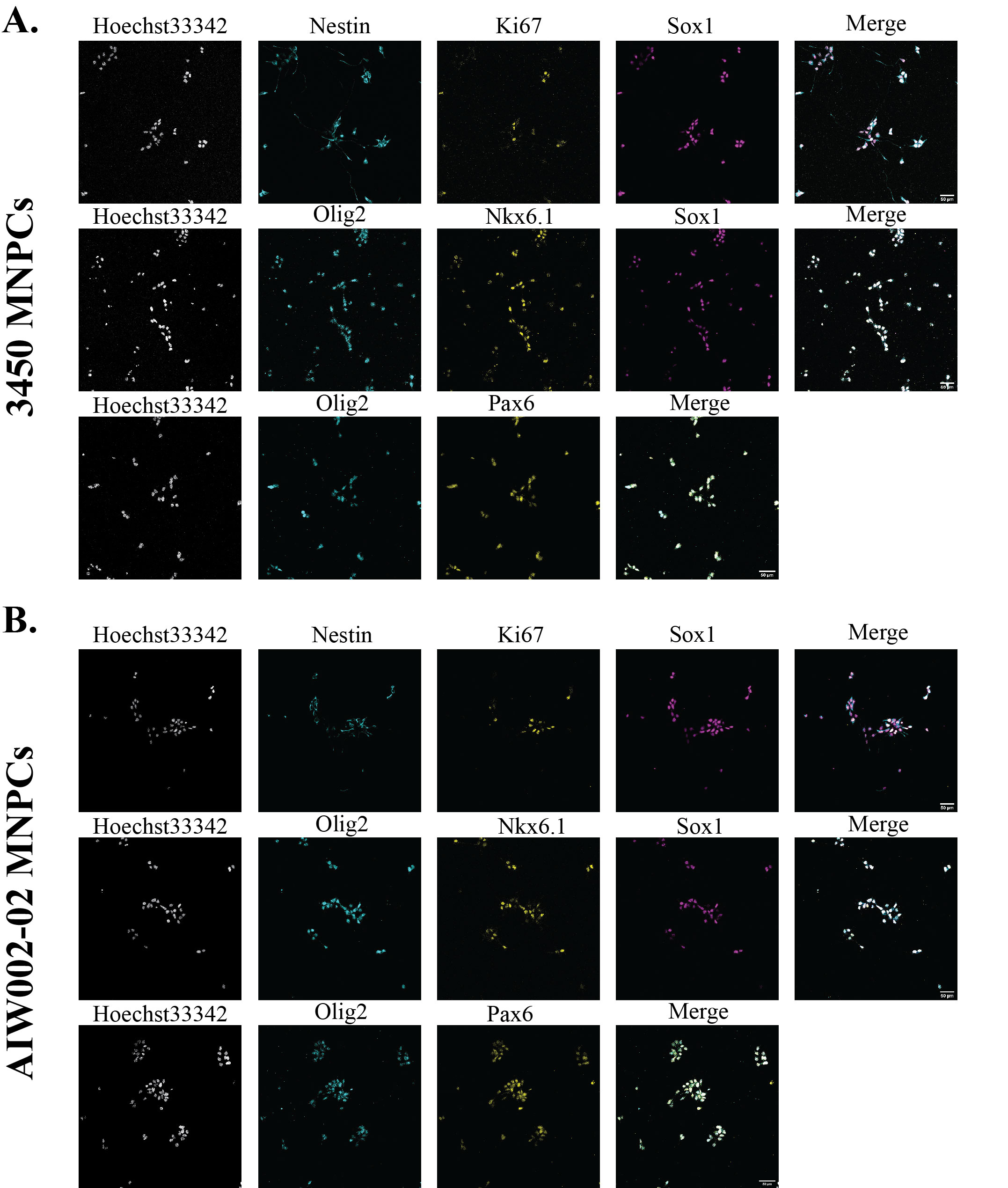

Supplement: Supplementary file 1 [file cells-12-00545-s001.zip › Supplementary Figure S1.jpg]

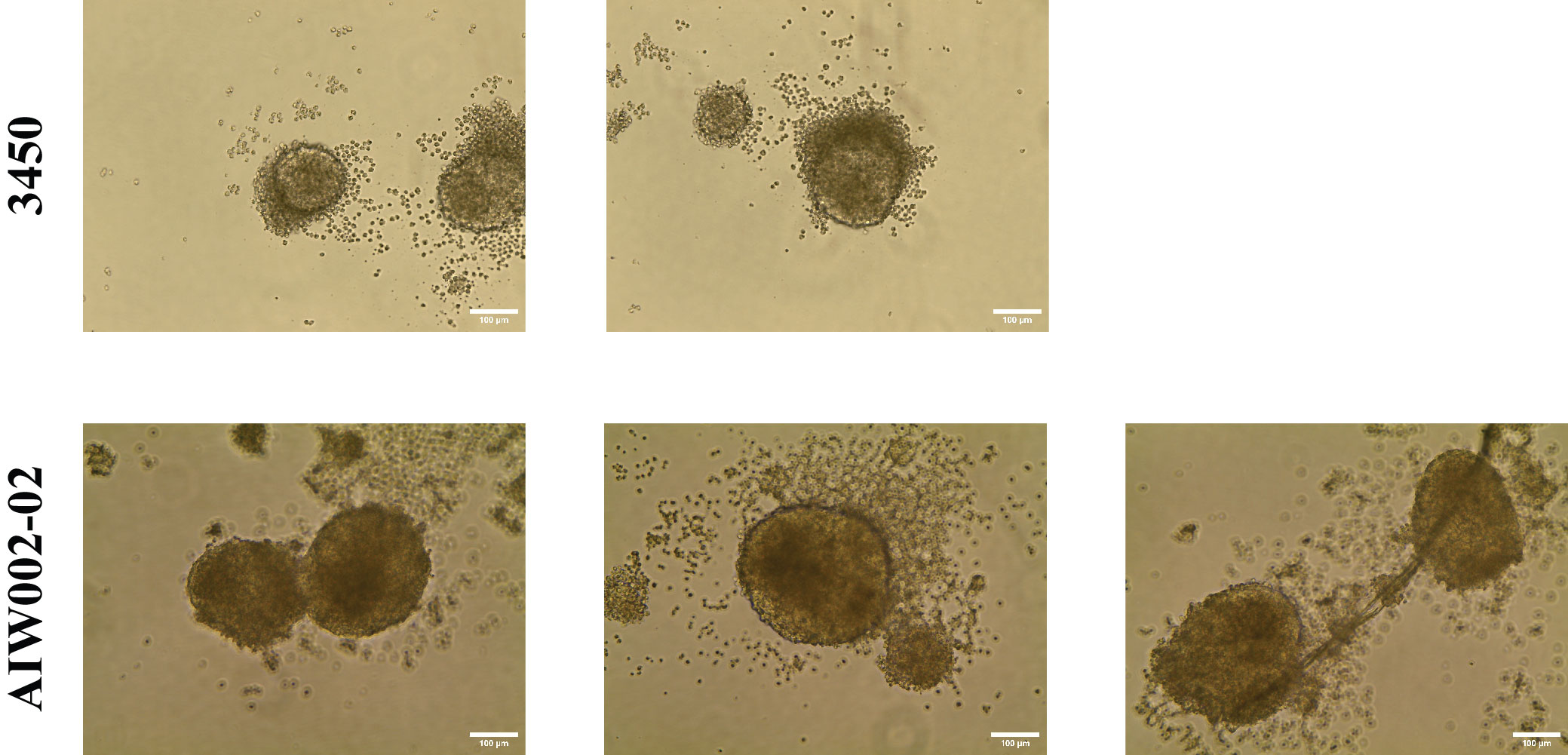

Supplement: Supplementary file 1 [file cells-12-00545-s001.zip › Supplementary Figure S2.jpg]

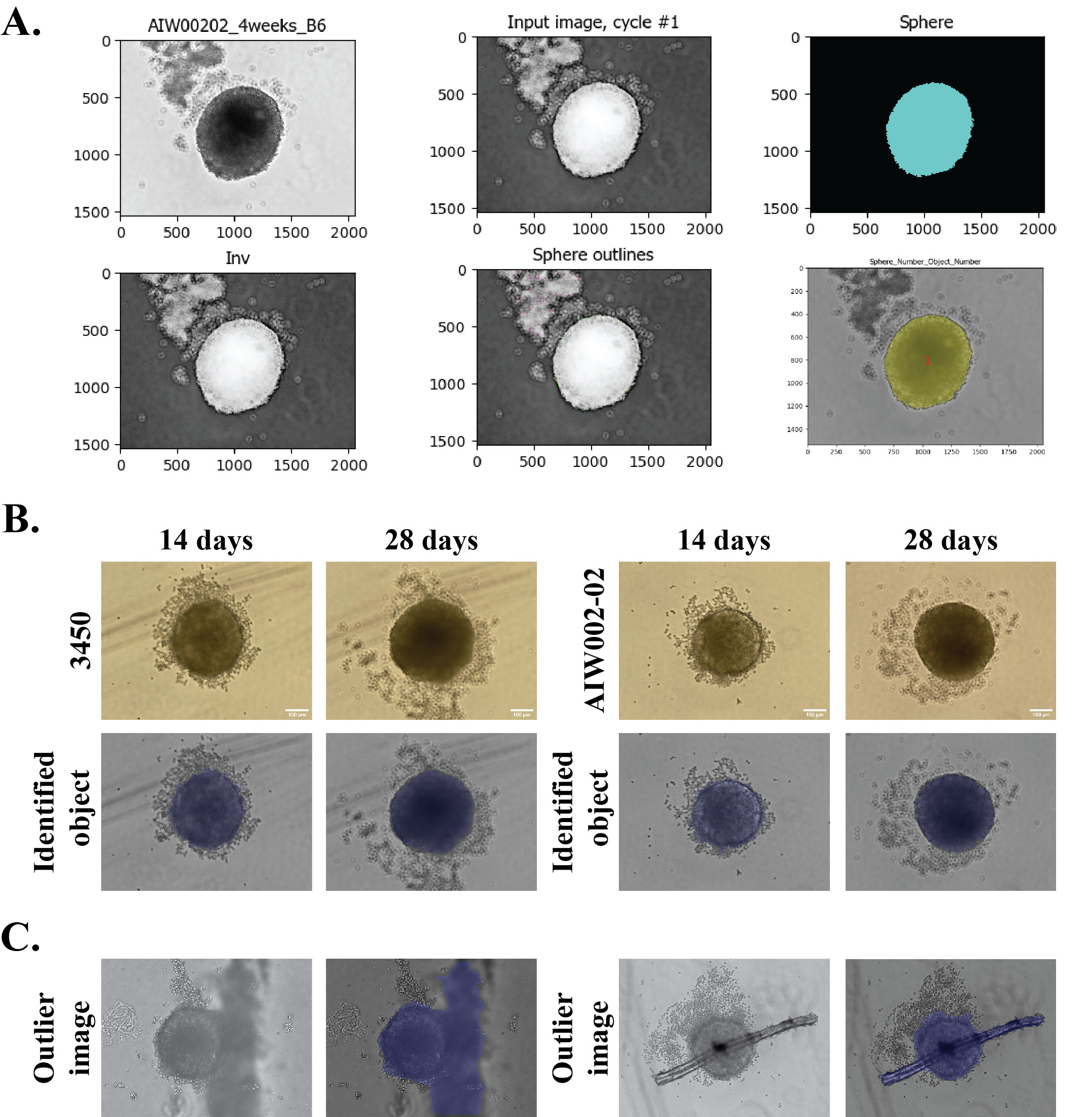

Supplement: Supplementary file 1 [file cells-12-00545-s001.zip › Supplementary Figure S3.jpg]

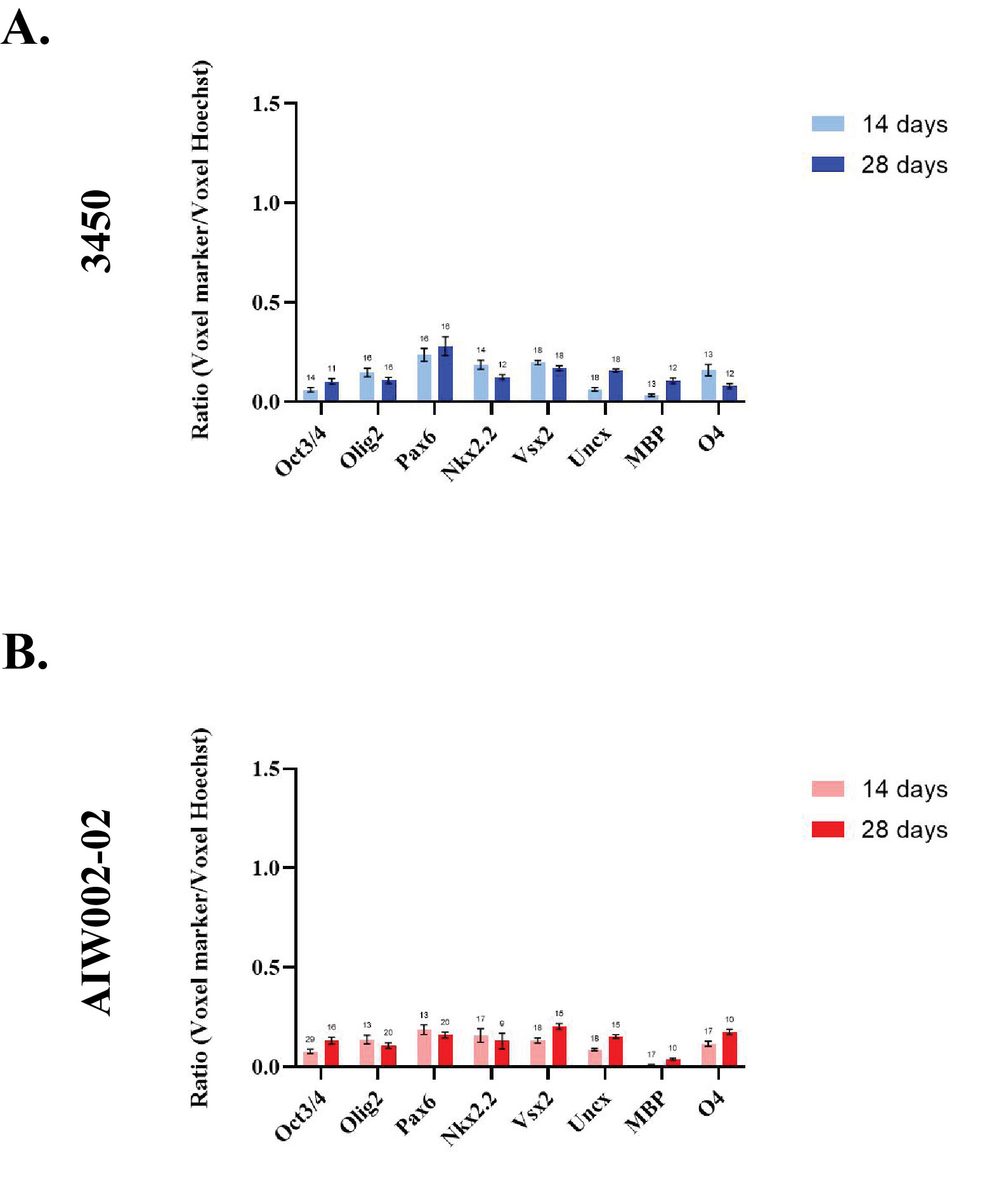

Supplement: Supplementary file 1 [file cells-12-00545-s001.zip › Supplementary Figure S4.jpg]
